# Identification of two metallothioneins in *Agaricus crocodilinus* reveals gene duplication and domain expansion, a pattern conserved across fungal species

**DOI:** 10.1007/s10534-025-00721-6

**Published:** 2025-07-18

**Authors:** Jan Sácký, Anna Chaloupecká, Jiří Šantrůček, Antonín Kaňa, Tereza Leonhardt, Jan Borovička, Pavel Kotrba

**Affiliations:** 1 Department of Biochemistry and Microbiology, University of Chemistry and Technology, Prague, Technická 3, 166 28 Prague 6, Czech Republic; 2 Institute of Geology, Czech Academy of Sciences, Rozvojová 269, 16500 Prague 6, Czech Republic; 3 Nuclear Physics Institute, Czech Academy of Sciences, Hlavní 130, 25068 Husinec-Řež, Czech Republic; 4 Department of Analytical Chemistry, University of Chemistry and Technology, Prague, Technická 5, 166 28 Prague, Czech Republic

**Keywords:** Gene duplication, Gene internal duplication, Heavy metals, *Agaricaceae*

## Abstract

*Agaricus crocodilinus* (*Agaricaceae*), an edible saprotrophic mushroom, accumulates high concentrations of cadmium (Cd) in unpolluted environments. This study investigates whether this species has evolved mechanisms to store Cd complexed with metallothioneins (MTs), proteins that bind heavy metal ions via cysteinyl (Cys)–thiolate bonds, how these MTs originated, and how similar mechanisms are present in other fungal species. Size exclusion chromatography revealed that a substantial fraction of Cd in *A. crocodilinus* sporocarps was sequestered in a 3.4 kDa complex containing Cys-rich peptides. Screening a sporocarp cDNA expression library in a Cd-sensitive *Saccharomyces cerevisiae* strain identified two MT transcripts, Ac*MT1* and Ac*MT2*, encoding 49-amino acid (AA) AcMT1 with 10 Cys and 32-AA AcMT2 with 7 Cys. The presence of AcMT2 in the 3.4 kDa Cd–peptide complex isolated from sporocarp was confirmed by mass spectrometry. In mycelial isolates exposed to heavy metals, Ac*MT1* was more strongly upregulated, while Ac*MT2* was more expressed under normal conditions. Sequence comparisons revealed that Ac*MT2* is closer to the ancestral gene, whereas Ac*MT1* is a more recent duplicate. Combined bioinformatic and functional evidence supports Ac*MT2* as a constitutively expressed MT involved in Cd binding in the sporocarp, while Ac*MT1*, though more inducible in mycelia and more protective in yeast, appears to serve a transient detoxification role. Moreover, the gene duplication and domain rearrangement mechanism underlying this MT diversification was also identified in other *Agaricales* and *Boletales* species.

**Supplementary Information:**

The online version contains supplementary material available at 10.1007/s10534-025-00721-6.

## Introduction

The ability of ectomycorrhizal and saprotrophic mushroom-forming fungi of the class *Agaricomycetes* (*Basidiomycota*) to accumulate a wide range of heavy metals in sporocarps has been known for decades. While the usual concentrations of Cd in common mushrooms range from ≤ 1 up to 5 mg kg^−1^ of dry tissue weight (dtw; Kalač, 2019), Cd concentrations in saprotrophic *Agaricus* species can be remarkably high, especially in *Agaricus crocodilinus* Murrill (Meisch et al. 1977; Melgar et al. 1998; Cocchi et al. 2006), where up to 249 mg Cd kg^−1^ dtw was reported (Cocchi and Vescovi 1997). Cadmium concentrations of 134–604 mg Cd kg^−1^ dtw have also been reported in another mushroom of the family *Agaricaceae*, *Cystoderma carcharias* (Borovička et al. 2019). However, these high levels in *C. carcharias* were only observed in a Cd-polluted area, whereas *A. crocodilinus* effectively accumulates Cd, and to some extent also Zn and Cu at unpolluted sites, like pastures and meadows, where it usually grows (Sácký et al. 2025).

The ability to accumulate Cd or other heavy metals intracellularly must be supported by respective biological mechanisms that protect the cells from heavy metal toxicity. In fungi, these may include deposition in sink compartments (particularly vacuoles) or immobilization, which may involve intracellular precipitation (e.g., with polyphosphates or carboxylates) or tight binding to cytosolic peptides (Bellion et al. 2006). Metallothioneins (MTs) are a heterologous group of such peptides, rich in cysteinyl residues (Cys; C; 13–35 mol% Cys) that provide heavy metal ions with a high affinity coordination environment almost exclusively with sulfur donor atoms from Cys in, for example, a tetrahedral geometry for divalent Zn^2+^ and Cd^2+^ and diagonal or trigonal geometry for monovalent Cu^+^ (Capdevila and Atrian 2011; Sutherland and Stillman 2011).

MTs are widespread across all life forms and play key roles in metal ion homeostasis, detoxification, and protection against oxidative stress (Sutherland and Stillman 2011; Ziller and Fraissinet-Tachet 2018). They vary greatly in size, from 22 amino acids in *Magnaporthe grisea* (Tucker et al. 2004) to over 300 in some mollusks and fungi, such as *Cryptococcus neoformans* (Ding et al. 2013; Palacios et al. 2014a) and *Tremella mesenterica* (Iturbe-Espinoza et al. 2016). Very long MTs typically arise through internal duplications of cysteine-rich motifs, increasing their metal-binding capacity. In fungi, many MTs have been linked to Cd, Zn, or Cu sequestration, often through yeast complementation studies or by observing metal-induced expression in mycelia. However, the genetic mechanisms underlying the Cys motif duplication remain poorly understood.

Since *A. crocodilinus* accumulates high levels of Cd, we hypothesized that MTs might contribute to metal detoxification in this species. Our initial observation that most Cd in wild-growing *A. crocodilinus* is bound in a Cd:MT complex led us to isolate and characterize two MT genes, AcMT1 and AcMT2. Their high sequence similarity pointed to a duplication event. To explore this further, we also examined the gene sequences of other MTs, which are reported in this study.

## Materials and methods

### Sporocarps and mycelial cultures of *Agaricus crocodilinus*

Sporocarps of *A. crocodilinus* were collected from a meadow in a rural area near the village of Morašice in Eastern Bohemia, Czech Republic. The procedures of sporocarp handling, including identification, mycelial isolate propagation (from collection B1081a containing 149 mg Cd kg^−1^ dtw, 255 mg Zn kg^−1^ dtw) and storage are described in Sácký et al. (2025). The mycelium of *A. crocodilinus* was maintained at 25 °C in the dark on potato dextrose broth (PD) agar plates containing 2 g L^−1^ potato extract (Formedium, UK) and 10 g L^−1^ glucose (Formedium, UK), and 10 g L^−1^ agar (Formedium, UK). The internal transcribed spacer 1 (ITS1) of the sporocarp and isolate were deposited in GenBank (OR467497.1).

### Zn and Cd protein-associated metal distribution in *Agaricus crocodilinus* sporocarps

Frozen sporocarps from collection B1081a were used in protein-associated metal distribution analysis. The collected sporocarps (5 g, fresh weight, stipes and caps together, in natural proportion) were disintegrated by using a OneShot Cell Disrupter device at 2 kbar (Constant Systems Ltd.). The protein extraction and size exclusion chromatography (SEC) on a Superdex Peptide 10/300 GL column (GE-Healthcare), with 50 mM HEPES, 25 mM KNO_3_ (pH 7.3) as a mobile phase, and inductively coupled plasma mass spectrometry (ICP-MS) detection of ^66^Zn and ^111^Cd isotopes followed the procedure of Sácký et al. (2022). Between the individual runs, the SEC column was regenerated with 5 mM EDTA and rinsed with 35 mL of the mobile phase. Ribonuclease A (13.7 kDa) and glutathione (0.3 kDa) were used as molecular mass standards.

Pooled SEC fractions 29–32 from the sporocarp eluate containing 3.4 kDa Cd and Zn complex were concentrated to final volume of 125 μL using a Microcon YM3 centrifugal filter device (Millipore). The ligands were labeled fluorescent in a reaction with a sulfhydryl-specific 7-fluorobenzofurazan-4-sulfonic acid (SBD-F; Sigma-Aldrich) and resolved by sodium dodecyl sulfate–polyacrylamide gel electrophoresis (SDS-PAGE) as described previously (Osobová et al. 2011). A rabbit MT1A (Sigma-Aldrich) was used as a molecular mass standard.

### Zn and Cd protein-associated metal distribution in *Agaricus crocodilinus* mycelia

Approximately 3 mm^3^ block of a 21-day-old colony from an agar plate was transferred to 20 mL of PD medium and grown for 28 days (stationary floating culture). The resulting mycelium was transferred to 25 mL of fresh PD medium supplemented with 5 µM Cd^2+^ or 250 µM Zn^2+^ and incubated for 3 days at 25 °C in the same manner. The metal-exposed mycelia (approximately 0.35 g fresh weight) were disintegrated by using a OneShot Cell Disrupter device at 2 kbar (Constant Systems Ltd.). The protein extraction and size exclusion chromatography (SEC) on a Superdex Peptide 10/300 GL column (GE-Healthcare), with 50 mM HEPES, 25 mM KNO_3_ (pH 7.3) as a mobile phase, and inductively coupled plasma mass spectrometry (ICP-MS) detection of ^111^Cd or ^66^Zn isotopes followed the procedure of Sácký et al. (2022). Between the individual runs, the SEC column was regenerated with 5 mM EDTA and rinsed with 35 mL of the mobile phase. Ribonuclease A (13.7 kDa) and glutathione (0.3 kDa) were used as molecular mass standards.

### Tandem mass spectrometry (MS/MS) analysis of ligands in Cd/Zn-peptide complex

Before mass spectrometry analysis, peptides were reduced with dithiothreitol (5 mM, 50 °C, 30 min), alkylated with iodoacetamide (25 mM, room temperature, 30 min, in dark) and finally cleaned up with ZipTip pipette tips containing C18 reverse phase according to manufacturer’s instructions (Merck Millipore). Mass spectra were acquired using a timsTOF HT mass spectrometer (Bruker Daltonics, Bremen, Germany) coupled with a nanoElute 2 liquid chromatograph (Bruker Daltonics, Bremen, Germany). Dry samples were resuspended in 50 µL of 3% (v/v) acetonitrile and 0.1% (v/v) formic acid. Mobile phase A consisted of water and 0.1% (v/v) formic acid, and mobile phase B consisted of acetonitrile and 0.1% formic acid. Samples (1 µL) were first loaded on a PepMap Neo-Trap column (300 μm × 5 mm, Thermo Scientific) at a pressure of 85 bar with 100% phase A for 10 min. Peptides were separated on a PepSep C18 analytical column (75 μm × 250 mm, Bruker Daltonics, Bremen, Germany) using an acetonitrile gradient as follows: 0–30 min. 3–35% B, 30–30.5 min. 35–95% B, 30.5–40 min. 95% B. Eluted peptides were introduced directly into a Captive spray 2 electrospray source (Bruker Daltonics, Bremen, Germany). Measurements were conducted in Data Dependent Analysis – Parallel Accumulation Serial Fragmentation mode. The mass range of the method was set to 100−1700 m/z. The ion mobility scan, expressed as the inverse value of reduced ion mobility, was performed in the range of 0.6 − 1.6 V s cm^−2^. The scan duration was 100 ms; ten ion mobility scans were performed between two MS spectra.

Peak lists were extracted from raw data by Data Analysis version 6.1 (Bruker Daltonics) and uploaded to the data management system Proteinscape (Bruker Daltonics). For protein identification, the Mascot server (version 2.4.1; Matrix Science) was used with a custom-made database containing Fungi portion of Swiss-prot database (downloaded from Uniprot website on the 12th September 2019) and expected peptides and complemented with common laboratory contaminants. The following parameters were set during searches: enzyme none, tolerance 10 ppm in MS mode and 0.05 Da in MS/MS mode, carbamidomethylation of cysteines was set as a fixed modification and oxidation of methionines as a variable modification, Mascot decoy search was used to calculate false discovery rate (FDR). Identified peptides/proteins were filtered so that final FDR was 1%.

### *Saccharomyces cerevisiae* metal-sensitive yeast strains

The genotypes of the metal-sensitive, URA3-deficient *S. cerevisiae* yeast strains used for heterologous expression of *A. crocodilinus* cDNAs in IC_50metal_ and plate assays are listed in Supplementary Table S1. The metal sensitivity of the *ycf1*Δ strain stems from the fact that as an ATP binding cassette (ABC) transporter, it is responsible for funneling heavy metals into the vacuole, thus its deletion renders the cells Cd-sensitive (Li et al. 1996). The Zn sensitivity of the *zrc1*Δ*cot1*Δ double mutant is due to the inactivation of its two CDFs, which transfer excess Zn into the vacuole (MacDiarmid et al. 2000). Deletion of *cup1* renders the mutant strain unable to produce the corresponding Cu-detoxification MT and makes the yeast particularly sensitive to Cu (Tamai et al. 1993). All yeast assays were performed in Synthetic Defined (SD) medium, prepared as follows: 6.7 g L^−1^ of yeast nitrogen base without amino acids, 20 g L^−1^ d-glucose, 50 mg L^−1^ adenine hemisulfate, and 30 mg L^−1^ each of l-histidine, l‑tryptophan, l-methionine, and l-leucine. Yeasts were grown in SD liquid or solid media at 30 °C.

### Construction and screening of cDNA expression library

For cDNA library construction, first, total RNA was isolated from 200 mg of freeze-dried sporocarp of *A. crocodilinus* using a RNeasy Plant Mini Kit (Qiagen) and its integrity was assessed by denaturing agarose gel electrophoresis. To obtain mRNA from total RNA, an Oligotex mRNA Mini Kit (Qiagen) was used. The cDNAs of *A. crocodilinus* transcripts were obtained from 100 ng of the sporocarp mRNA by using a Template Switching RT Enzyme Mix (New England Biolabs) following the manufacturer’s 2nd Strand cDNA Synthesis Protocol. In‑Fusion HD Cloning Kit (Takara) was used to insert the resulting cDNAs into 2μ yeast expression shuttle vector p426GPD (Mumberg et al. 1995) linearized by Q5 High-Fidelity DNA Polymerase (New England Biolabs). Primers used for the linearization are listed in Supplementary Table S2. The in vitro constructed cDNA library was transformed into NEB 10‑beta Competent *E. coli* cells, grown at 37 °C on Luria–Bertani (LB) agar supplemented with 150 μg mL^−1^ ampicillin, yielding 6 × 10^4^ of individual transformant colonies, which were washed from the plates with LB medium, pooled, and stored in 20% glycerol (v/v) at −80 °C. To obtain plasmids harboring the cDNAs a NucleoSpin Plasmid Kit (Macherey–Nagel) was used, and the resulting cDNA library was used to transform the *ycf1*Δ yeast strain. The yeast transformants were selected on *URA*^*−*^ selective SD medium plates (approximately 1 × 10^5^ single colonies were obtained), washed from the plates with fresh SD medium, and plated on SD agar plates supplemented with otherwise inhibitory 100 μM Cd^2+^. From the Cd tolerant colonies, plasmids were isolated by NucleoSpin Plasmid Kit (Macherey–Nagel), reamplified in *E. coli* DH5α and sequenced by using sequencing primers (Supplementary Table S2).

### Complementation assays in *Saccharomyces cerevisiae*

For yeast complementation assays, Ac*MT* cDNAs were amplified from sporocarp cDNA. Total RNA was isolated as described above, and 1 μg was reverse-transcribed using the High Capacity cDNA Reverse Transcription Kit (Applied Biosystems). The resulting cDNA served as a template for PCR using Q5 High-Fidelity DNA Polymerase (New England Biolabs) and primers targeting the 5′ and 3′ ends of the respective cDNA library clones (primer sequences are listed in Supplementary Table S2). PCR products were cloned into BamHI-digested p426GPD vector using the In-Fusion HD Cloning Kit (Takara) and transformed into *E. coli* DH5α. Plasmids were extracted with the NucleoSpin Plasmid Kit (Macherey–Nagel) and verified by Sanger sequencing (Eurofins Genomics) on both strands to confirm correct insertion. Resulting plasmids were transformed into the abovementioned yeast strains by the LiAc/SS-DNA/PEG method (Gietz and Schiestl et al. 2007). Transformed colonies were selected and maintained on SD medium without uracil to ensure retention of the plasmid DNA.

Metal tolerance plate assays were performed as follows. SD agar plates without metal addition or supplemented with Cd^2^⁺ (up to 75 μM), Cu^2^⁺ (up to 100 μM), or Zn^2^⁺ (up to 400 μM) were used to spot 4 μL of tenfold serial dilutions of mid-log *S. cerevisiae* strains cultures of OD₅₉₀ = 0.1 transformed with the p426GPD-derived plasmids. Plates were incubated for 24–48 h at 30 °C until the differences in growth inhibition could be clearly assessed.

To determine the 50% inhibitory concentrations of metals (IC₅₀_metal_) in liquid SD medium, *S. cerevisiae* strains were inoculated at OD₅₉₀ = 0.1, exposed to increasing metal concentrations, and their growth (as turbidity) was measured after 24 h (*ycf1*Δ and *zrc1*Δ*cot1*Δ) or 48 h (*cup1*Δ). Each IC₅₀_metal_ experiment was performed in triplicate, and the IC₅₀_metal_ values were calculated by fitting the data to the logistic function T(c) = T(0)/{1 + exp[(c − IC₅₀_metal_)/b]} using the Solver add-in in Microsoft Excel (Anton et al. 2004). One-way ANOVA followed by Tukey’s post hoc test (p < 0.05), was carried out in R using the aov() and TukeyHSD() functions (R Core Team 2022).

### Amplification of AcMT genes from genomic DNA, gene analyses, phylogenetic analyses

Genomic clones of Ac*MT*s were amplified from the total DNA isolated from freeze-dried sporocarp tissue using a NucleoSpin Plant II kit (Macherey–Nagel) by PCR using GoTaq polymerase (Promega) and primers targeting flanking untranslated sequences (Supplementary Table S2). Purification of the PCR products was performed with a NucleoSpin Gel and PCR clean up kit (Macherey–Nagel) and amplicons were sequenced by Sanger sequencing (Eurofins Genomics). The resulting genomic sequence data were deposited in GenBank under the accession numbers PP780932 (Ac*MT1*) and PP780933 (Ac*MT2*).

The protein sequences deduced from the Ac*MT* cDNAs were confirmed by a BLASTp analysis against the UniProtKB/Swiss-Prot database, and the retrieved characterized *Agaricomycetes* MTs were aligned with AcMTs in ClustalW (Larkin et al. 2007).

A phylogenetic tree was constructed using the Neighbor-Joining method (Saitou and Nei 1987) implemented in MEGA X, with default parameters (Kumar et al. 2018). The tree was rooted using *S. cerevisiae* CUP1-1 as an outgroup and evaluated by bootstrap analysis with 10,000 replicates to assess branch support (Felsenstein 1985). The evolutionary distances were computed using the Poisson correction method (Zuckerkandl and Pauling 1965) and are in the units of the number of amino acid substitutions per site.

### Relative quantification of Ac*MT*s

The expression response of Ac*MT*s to metal exposure was evaluated using quantitative reverse transcription PCR (qRT-PCR). Approximately 3 mm^3^ blocks of 21-day-old fungal mycelia were inoculated into 500 mL flasks containing 20 mL of PD medium and incubated under stationary conditions in the dark for 20 days to allow for mycelial growth. During incubation, the mycelia formed floating mats on the surface of the medium. Following this period, cultures were transferred to fresh PD medium (20 mL) supplemented with Cd^2^⁺, Zn^2^⁺, or Cu^2^⁺ and incubated for an additional 24 h at the same conditions. A parallel culture maintained in metal-free PD medium served as the control. Total RNA was extracted, treated with RNase-Free DNase (Qiagen) to eliminate genomic DNA, and reverse transcribed into cDNA using the High-Capacity cDNA Reverse Transcription Kit (Applied Biosystems), starting from 1 μg of RNA. The β-tubulin gene Ac*TUB2* (GenBank accession no. PV055698) served as an internal control for normalization. PCR amplification efficiencies were calculated as 98% for Ac*MT1*, 86% for Ac*MT2*, and 89% for Ac*TUB2* (primers in Supplementary Table S2). Relative gene expression was calculated using the 2^−ΔΔCt^ method (Pfaffl 2001). All experiments were conducted in three independent biological replicates, each performed in technical duplicate. One-way ANOVA followed by Tukey’s post hoc test (p < 0.05), was carried out in R using the aov() and TukeyHSD() functions (R Core Team 2022).

## Results

### Metal distribution in the sporocarps

**Fig. 1:**
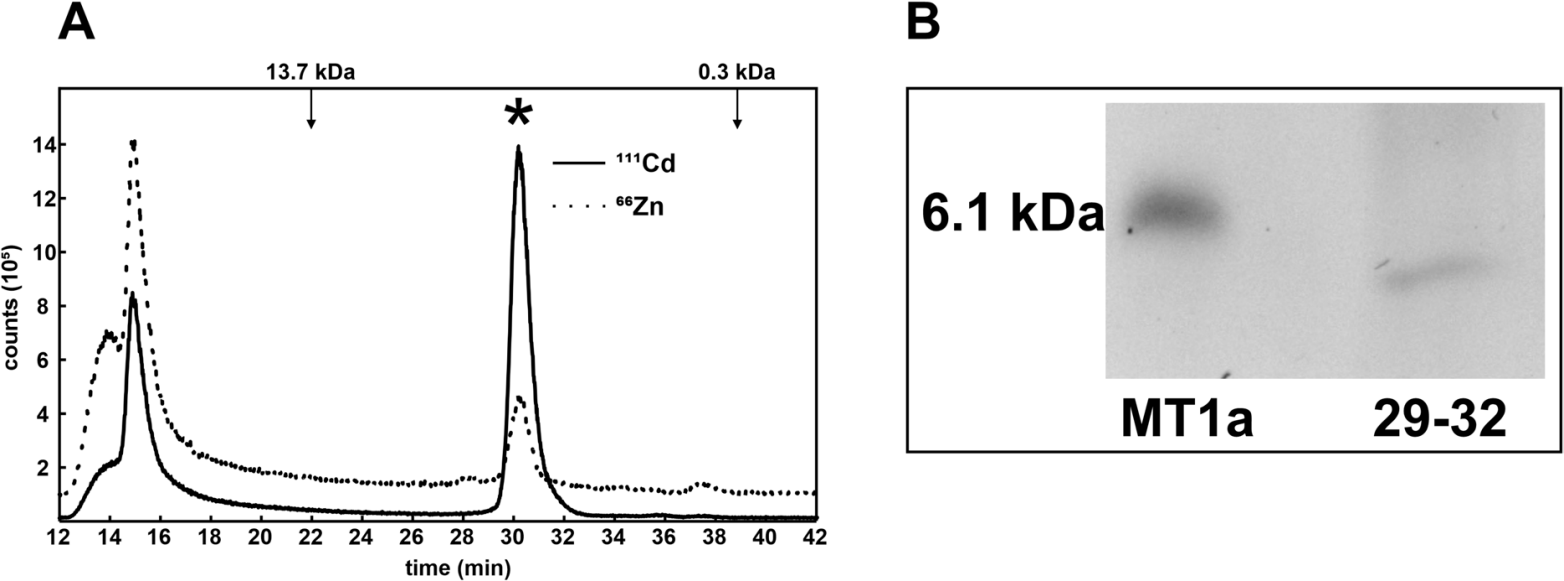
Metal distribution and characterization of SEC fractions from *Agaricus crocodilinus* sporocarp. **A** Distribution of metals in cell-free extracts analyzed by size exclusion chromatography. **B** Electropherogram showing the protein content of the metal-containing fractions 29–32. The sporocarp extract was fractionated by SEC, and the concentrations of Cd (solid line) and Zn (dotted line) in the eluate were monitored by ICP-MS using the ^111^Cd and ^66^Zn isotopes. The elution maxima of molecular mass standards are indicated by arrows; the 3.4 kDa metal–peptide complex is marked with an asterisk. Panel B shows an electrophoreogram of fluorescence-labeled Cys-containing peptides present in the 3.4 kDa metal–peptide complex, alongside a 6.1 kDa rabbit MT1A standard used for size comparison

To gain an initial insight into the peptide-associated distribution of metals in *A. crocodilinus*, the sporocarp extract was separated by size exclusion chromatography (SEC). It was shown that a substantial part of the intracellular Cd (47%) and some Zn (11%) was eluted in a peak at 29–32 min with a maximum equal to approximately 3.4 kDa (Fig. 1A). In addition to the abundant 3.4 kDa metal-protein complex high amounts of Cd and Zn eluted from the SEC column (exclusion limit of 20 kDa) in early fractions with the molecular mass of > 13.7 kDa. The > 13.7 kDa peak is formed due to the low resolution of the column at this volume point and can contain both naturally metalated proteins (e.g. enzymes) or can be an artifact stemming from non-specific metalation of proteins with the metal ions released during the extraction from cellular compartments. No Zn or Cd was eluted in the late fractions (< 0.3 kDa) and no Zn or Cd peak at 24–26 min, equal to approximately 10 kDa, was detected.

Considering a cysteinyl-containing peptide as a possible ligand in the 3.4 kDa complex, the corresponding sporocarp fractions 29–32 were pooled, derivatized with Cys-labeling SBD-F and resolved by SDS-PAGE. As shown in Fig. 1B, the complex contained putative cysteinyl-containing peptides with an estimated molecular mass less than 6.1 kDa.

Since protein sequencing is not possible at our facility, a different approach for the determination of the peptides within this 3.4 kDa fraction was used.

### Metal distribution in the mycelial isolate

**Fig. 2:**
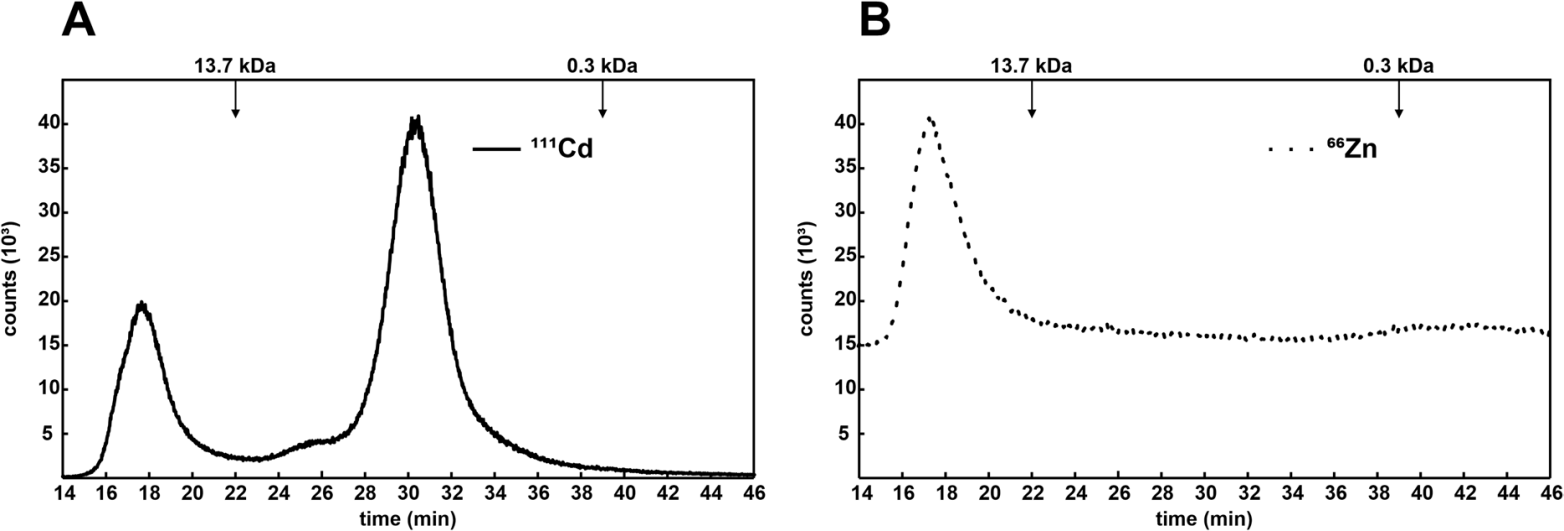
Metal distribution analysis of cell-free extracts from *Agaricus crocodilinus.* Mycelia were grown in the presence of 5 µM Cd^2+^ (**A**) or 250 µM Zn^2+^ (**B**). Mycelial extracts were fractionated by size-exclusion chromatography (SEC), and the concentrations of Cd (solid line) or Zn (dotted line) in the eluates were monitored by inductively coupled plasma mass spectrometry (ICP-MS) using the ^111^Cd and ^66^Zn isotopes. Elution maxima of molecular mass standards are indicated by arrows; the 3.4 kDa metal–peptide complex is marked with an asterisk. A small Cd peak, likely mycophosphatin, was formed between 24 and 26 min

To assess in vivo formation of metal–peptide complexes, *A. crocodilinus* mycelial cultures were exposed to 5 µM Cd^2^⁺ or 250 µM Zn^2^⁺ for 3 days. The treated mycelia were harvested, mechanically disrupted, and the resulting cell-free extracts were subjected to SEC–ICP–MS. As shown in Fig. 2A, exposure to Cd^2^⁺ resulted in the formation of a defined Cd–peptide complex with an apparent molecular mass of approximately 3.4 kDa, consistent with the complex previously detected in the sporocarp. Furthermore, a small peak, likely mycophosphatin, at 24–26 min, was detected. In contrast, Fig. 2B shows that no analogous Zn complex was detected in Zn-exposed mycelia, despite the high concentration of Zn^2^⁺ used in the treatment.

### Identification of cDNA sequences of putative MTs from *Agaricus crocodilinus* sporocarp

To discern the DNA sequence of the peptide in the Cd/Zn-peptide complex, a cDNA library screening in *S. cerevisiae* Cd-sensitive yeast mutant *ycf1*Δ was performed. This strain is unable to grow on SD agar plates supplemented with ≥ 25 μM Cd^2+^, thus 100 μM Cd^2+^ was selected as the threshold for selecting possible colonies containing the most likely Cd-protective transcripts. The p426GPD-based cDNA expression library was constructed from sporocarp tissue to ensure naturally relevant transcripts would be acquired. Plating approximately 1 × 10^5^ *S. cerevisiae ycf1*Δ colonies harboring the *A. crocodilinus* cDNA library on SD agar with 100 μM Cd^2^⁺ yielded over 50 Cd-tolerant transformants. These contained either of two cDNAs with open reading frames of 150 or 99 nucleotides, encoding 49- and 32-amino-acid peptides, respectively. The two peptides were designated AcMT1 and AcMT2, respectively, and were compared with characterized metallothioneins from *Agaricomycetes* known to function in metal detoxification. The comparison revealed a substantial level of similarity to many known fungal MTs, especially those implied in Cd and Cu complexation.

### Identification of peptides in the 3.4 kDa Cd/Zn-peptide complex from* Agaricus crocodilinus* sporocarp by mass spectrometry

The objective of this experiment was to determine whether the 3.4 kDa metal–peptide complex observed in the sporocarp extract contains the AcMT2 peptide identified through cDNA library screening. To this end, fractions 29–32 from the SEC run, corresponding to the Cd/Zn peak, were pooled and subjected to mass spectrometry (MS) analysis. The analyzed spectra were compared to a custom fungal protein database including the sequences of AcMT1 and AcMT2. The analysis confirmed the presence of peptide fragments corresponding to AcMT2 in these fractions (Supplementary Fig. S1), with AcMT2 being the most probable peptide detected, thus demonstrating that AcMT2 is a constituent of the observed 3.4 kDa Cd/Zn-peptide complex.

### Protein sequence analysis and gene duplication analysis

**Fig. 3:**
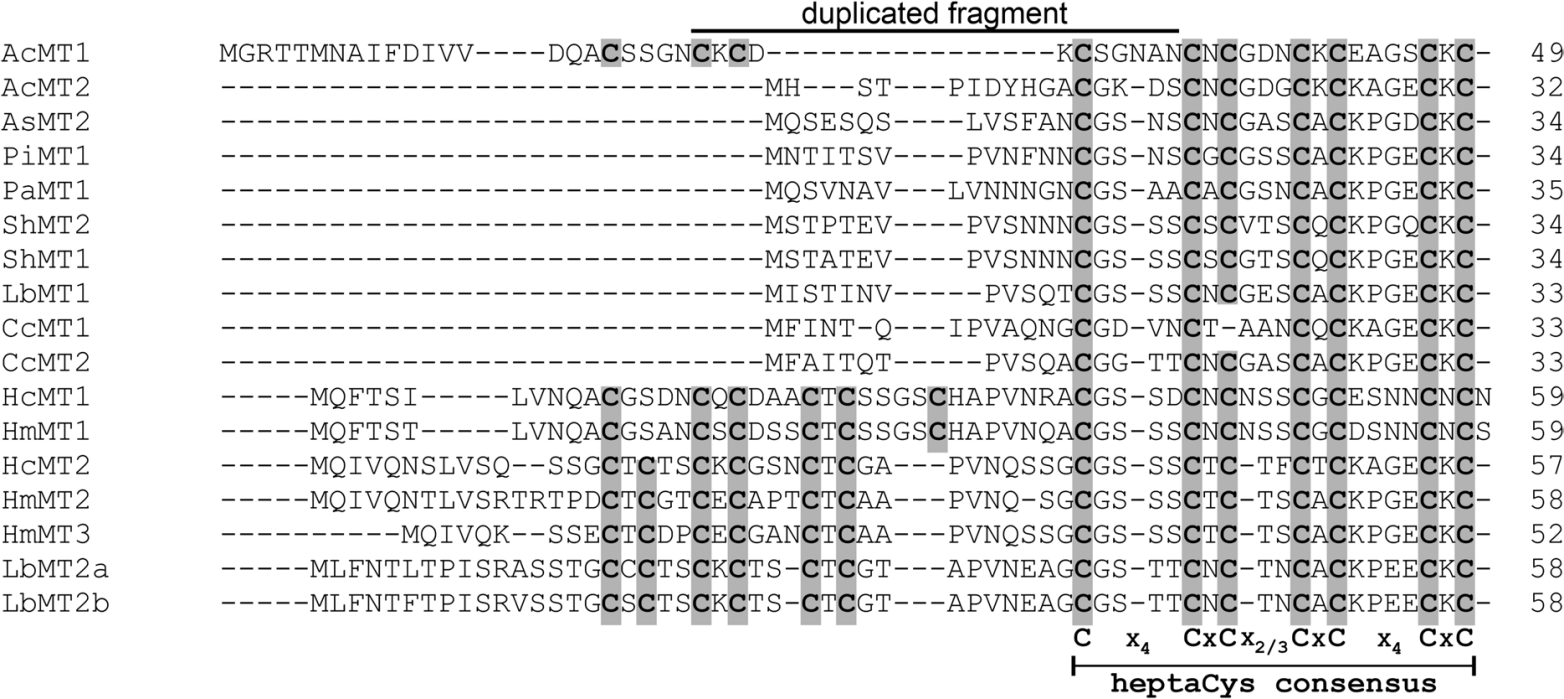
Amino acid alignment of *A. crocodilinus* AcMTs with characterized fungal MT homologues. Cysteinyl residues are highlighted in grey and the conserved heptaCys motif is marked below the alignment. Duplicated fragment of AcMT1 is marked above the alignment. Aligned AcMT homologues (GenBank accession numbers): AsMT2 (AGO04615), PiMT1 (AAS19463), PaMT1 (AJO67962), ShMT1/2 (AUS94322/AUS94321), LbMT1/2a/2b (AHI43933/AHI43934/AHI43935), CcMT1/2 (QNN26299/QNN26300), HcMT1/2 (ACJ65191/ACJ65192), HmMT1/2/3 (AHA31878/AHA31879/AHL29949)

**Fig. 4:**
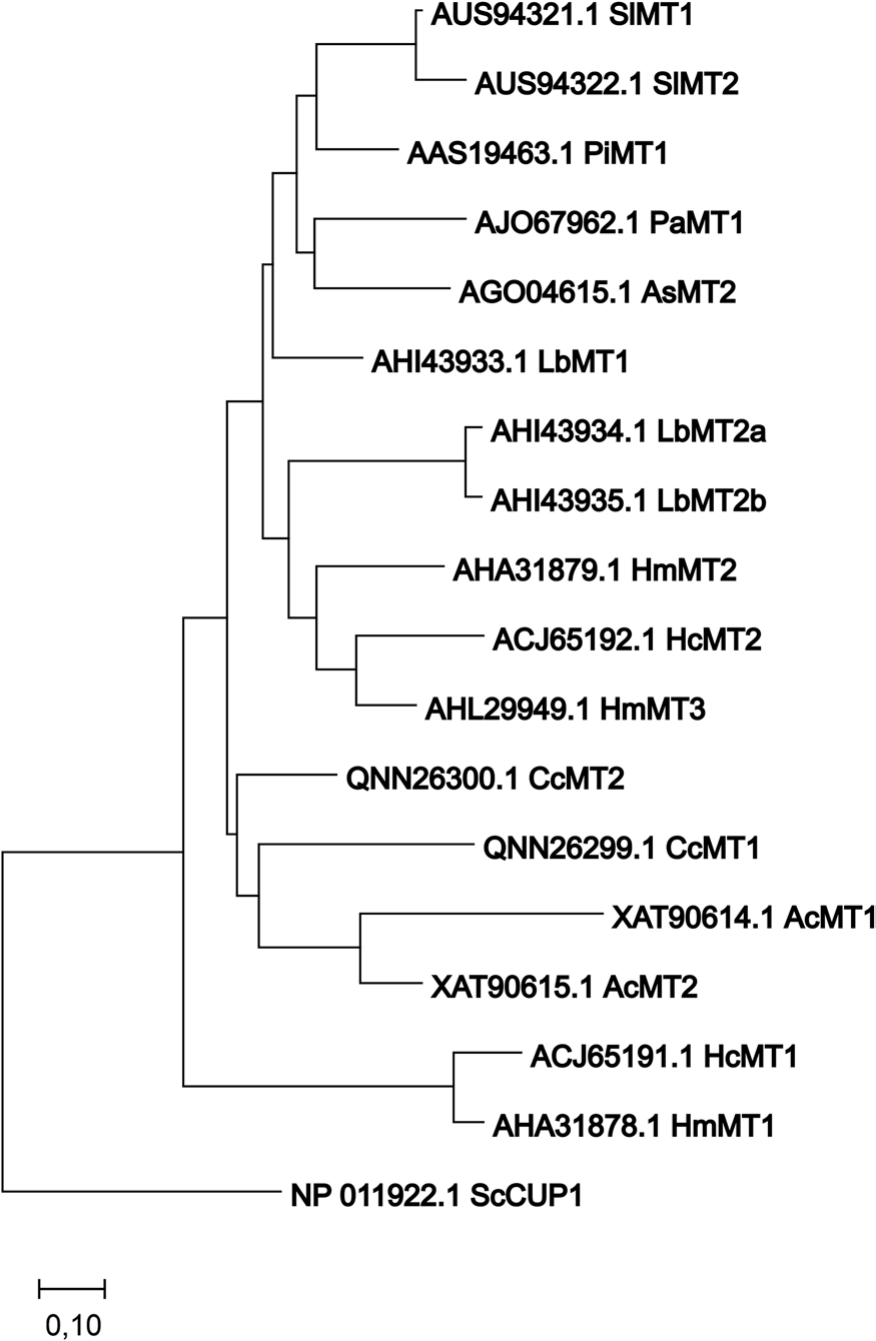
Phylogenetic tree of fungal MTs with known function in metal detoxification. The tree was constructed using Neighbor-joining method, with 10,000 bootstraps, support values over 60 are displayed. Protein sequences for the alignment were obtained from GenBank and their identifiers and names are displayed. Branch lengths represent evolutionary distances measured in substitutions per site. Scale bar indicates 0.10 substitutions per site. The tree is rooted using *Saccharomyces cerevisiae* CUP1-1 metallothionein as an outgroup

As documented in Fig. 3, the hallmark feature of AcMT orthologs, which include both short MTs (up to 35 AA) and long MTs (up to 59 AA), is a highly conserved C-terminal heptaCys C-x_4_-C-x-C-x_2,3_-C-x-C-x_4_-C-x-C motif (x represents any AA). This motif is preserved in AcMT2, whilst AcMT1 showed between the first two Cys an unusual spacing of C-x_5_-C, caused by an extra AA residue. Irrespective of this difference, AcMTs within their heptaCys motifs shared 70% identity with each other, which suggested an idea of Ac*MT*s being duplicate genes with a common predecessor. The close phylogenetic relationship of AcMT1 and AcMT2 was also underscored by a phylogenetic tree (Fig. 4), in which these two MTs clustered together with high support. AcMT1 branch is longer, suggesting it is evolutionary further from the common ancestor, while AcMT2, having a shorter branch, was under strong selective pressure, retaining the original ancestral function.

**Fig. 5:**
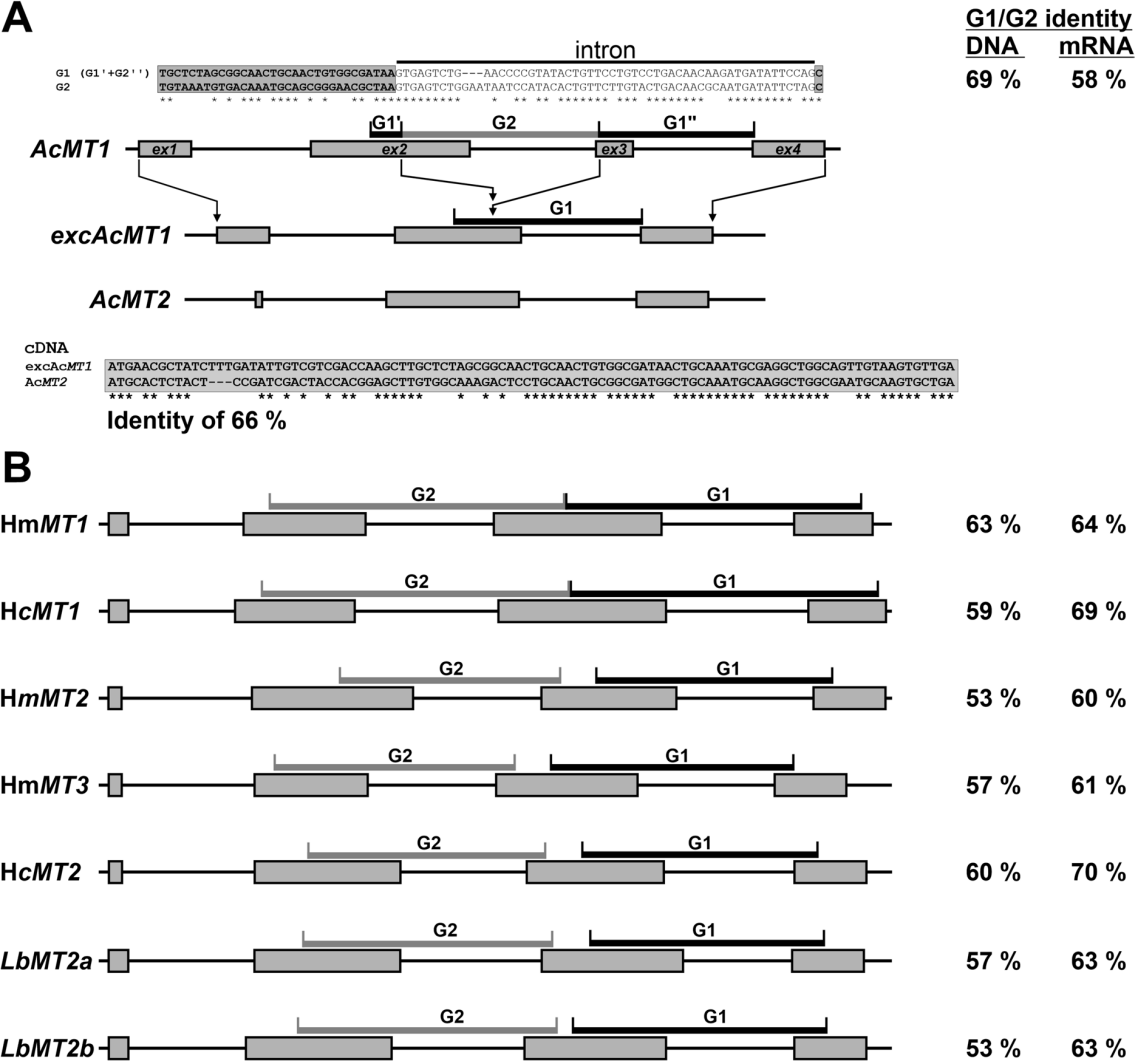
Internal duplications in AcMT1 and other Agaricomycete MTs. **A** and **B** show schematic representations of proposed internal duplication events in Ac*MT1* and in other long *Agaricomycete* MT homologues, respectively. Duplicate fragments are labeled G1 and G2, with their nucleotide (DNA or mRNA) identity indicated as % G1 to G2. Exons (*ex*) are shown as grey rectangles. In panel A, exon organization is compared between Ac*MT2* and the computationally derived ancAc*MT1* (Ac*MT1* lacking G2). cDNA sequences of Ac*MT2* and ancAc*MT1* are aligned below, with identical nucleotides marked by asterisks

To gain a better insight into the evolutionary relationship between Ac*MT1* and Ac*MT2*, the corresponding genomic sequences were amplified for comparison. The mRNA-to-genomic sequence alignments revealed that the Ac*MT1* and Ac*MT2* genes contained four and three exons, respectively, interrupted by small, about 50 bp introns (Fig. 5A). Furthermore, a closer look at the genomic Ac*MT1* sequence suggested that its extension was due to an internal gene duplication with Ac*MT1* undergoing further modification that extended its sequence with an additional Cys-containing domain.

It appears that the internal duplication in Ac*MT1* involved an insertion of replication fragment G1, which, after duplication and self-insertion, created fragment G2 (Fig. 5A). Fragment G2 covers a part of exon 2 with a downstream intron plus the first base pair of exon 3, inserted into what would have been the exon 2 of a hypothetical ancestral gene ancAc*MT1*. As a result, the assumed internal duplication event has added a stretch coding for C-x-C-x_2_-C-x_5_ (Fig. 3). The *in silico* excision of the G2 coding sequence in the cDNA of Ac*MT1* creates a hypothetical ancAc*MT1* transcript that shares a high degree of identity with that encoding AcMT2 and changes the Cys spacing in the heptaCys of hypothetical ancAcMT1 sequence to the conserved heptaCys C-x_4_-C-x-C-x_3_-C-x-C-x_4_-C-x-C consensus motif (Fig. 3).

To see if the scenario of internal duplication holds for other MTs a similar in silico excision and comparison of gene sequences was done also for long homologues of AcMTs (original data in Supplementary Data S1). This analysis revealed, that in the case of the long type of MTs, the genes were extended by a more “perfect” tandem insertion without disturbing the heptaCys-coding sequence (Fig. 5B).

### Functional complementation assay in metal-sensitive *Saccharomyces cerevisiae* yeast strains

To confirm the metal binding capacity of AcMTs in yeast model their corresponding full-length cDNAs were constitutively expressed in metal-sensitive *S. cerevisiae* strains *ycf1*Δ, *zrc1*Δ*cot1*Δ, or *cup1*Δ. The yeast strains transformed with empty expression vector p426GPD served as controls. With these transformed yeasts, two types of assays were performed, spot plate assay and IC_50metal_ assay.

**Fig. 6:**
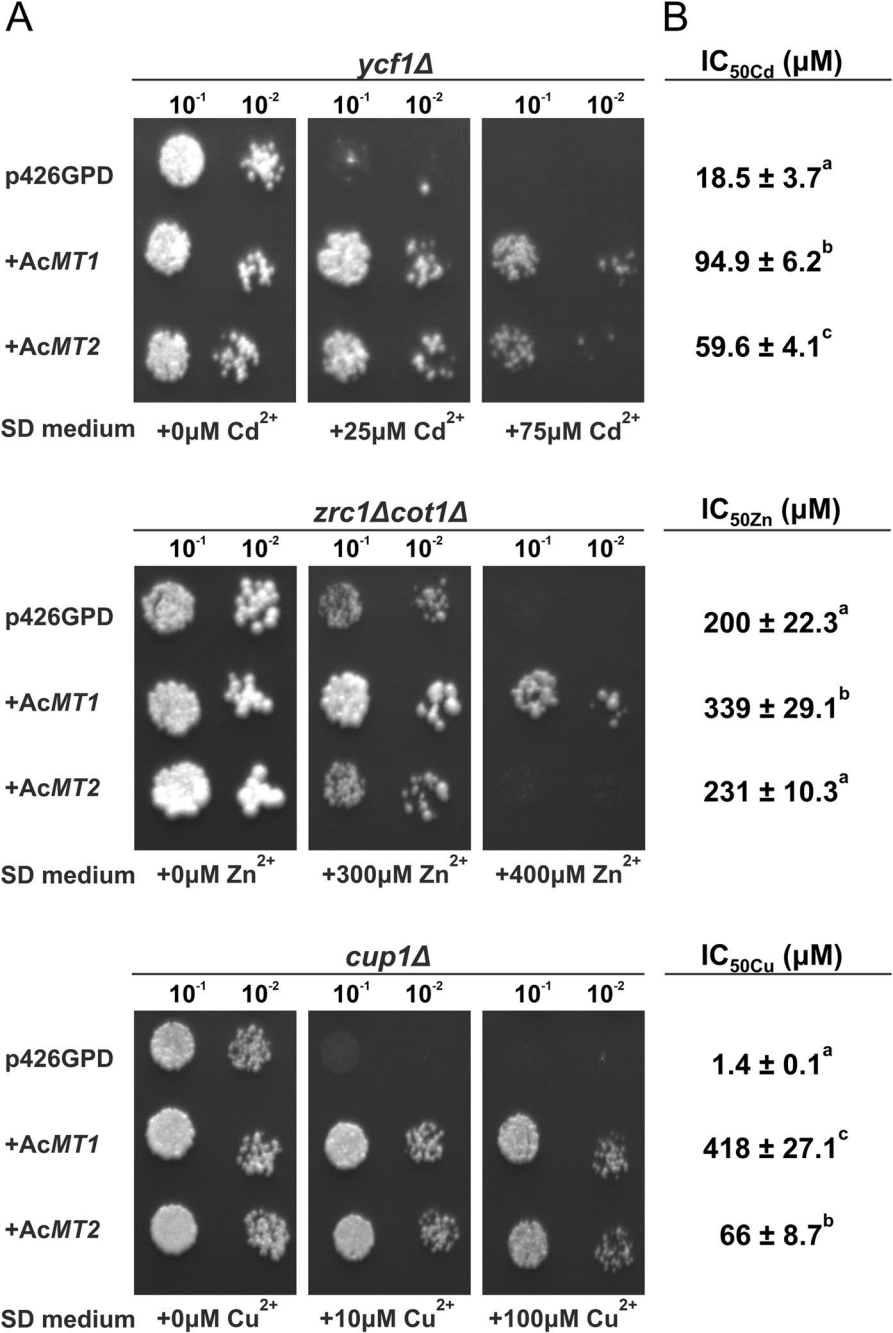
Metal tolerance in *S. cerevisiae* expressing Ac*MT*s. Tolerance to Cd, Zn, and Cu was assessed in metal-sensitive *S. cerevisiae* mutants (*ycf1*Δ, *zrc1*Δ*cot1*Δ, and *cup1*Δ) transformed with the p426GPD vector carrying either no insert or the Ac*MT1* or Ac*MT2* cDNA, as indicated. Shown are results from spot plate assays (left) on SD agar with or without the indicated metal supplements, and corresponding metal dose-dependent growth inhibition in liquid media (right), expressed as IC₅₀ₘₑₜₐₗ values. The IC₅₀ values represent means ± standard deviation from at least three independent biological replicates. Different letters in superscript denote statistically significant differences (one-way ANOVA with Tukey’s post hoc test, p < 0.05). For turbidity data, see Supplementary Figure S2

As can be seen from the spot plate assays (Fig. 6A) both Ac*MT1* and Ac*MT2* protected the *ycf1*Δ strain from cadmium poisoning at more than 75 µM Cd^2+^, while the control, was no longer viable at more than 25 µM Cd^2+^. Similarly, both Ac*MT1* and Ac*MT2* protected the *cup1*Δ strain from copper poisoning at more than 10 µM Cd^2+^. Regarding zinc poisoning, only the longer Ac*MT1* had a protective effect on the *zrc1*Δ*cot1*Δ strain.

These preliminary findings were corroborated by IC₅₀ determinations in liquid media (Fig. 6B and Supplementary Figure S2). Ac*MT1* consistently conferred greater metal tolerance than Ac*MT2* across all tested mutant strains. In *ycf1Δ*, Ac*MT1* expression resulted in a ~ fivefold increase in IC₅₀ compared to the p426GPD-only control, while Ac*MT2* conferred a ~ threefold increase. In *cup1Δ*, Ac*MT1* had a dramatic effect, raising the IC₅₀ nearly 300-fold, whereas Ac*MT2* provided a ~ 50-fold increase. In the case of *zrc1Δcot1Δ*, only Ac*MT1* conferred a statistically significant increase in zinc tolerance, though modest, just under twofold, while Ac*MT2* showed no meaningful improvement over the control. It can be also seen from Supplementary Figure S2, that the protection against Zn poisoning was visible only in the 200 µM–400 Zn^2+^ µM range, suggesting only a very weak Zn-protective effect of the Ac*MT*s. This is also reflected on the spot plate assays, where at 400 Zn^2+^ µM Ac*MT1* displays relatively good protective effect, but overall, the Ac*MT*s do not differ from empty vector control.

### Induction* of AcMTs* in metal stress condition

**Fig. 7:**
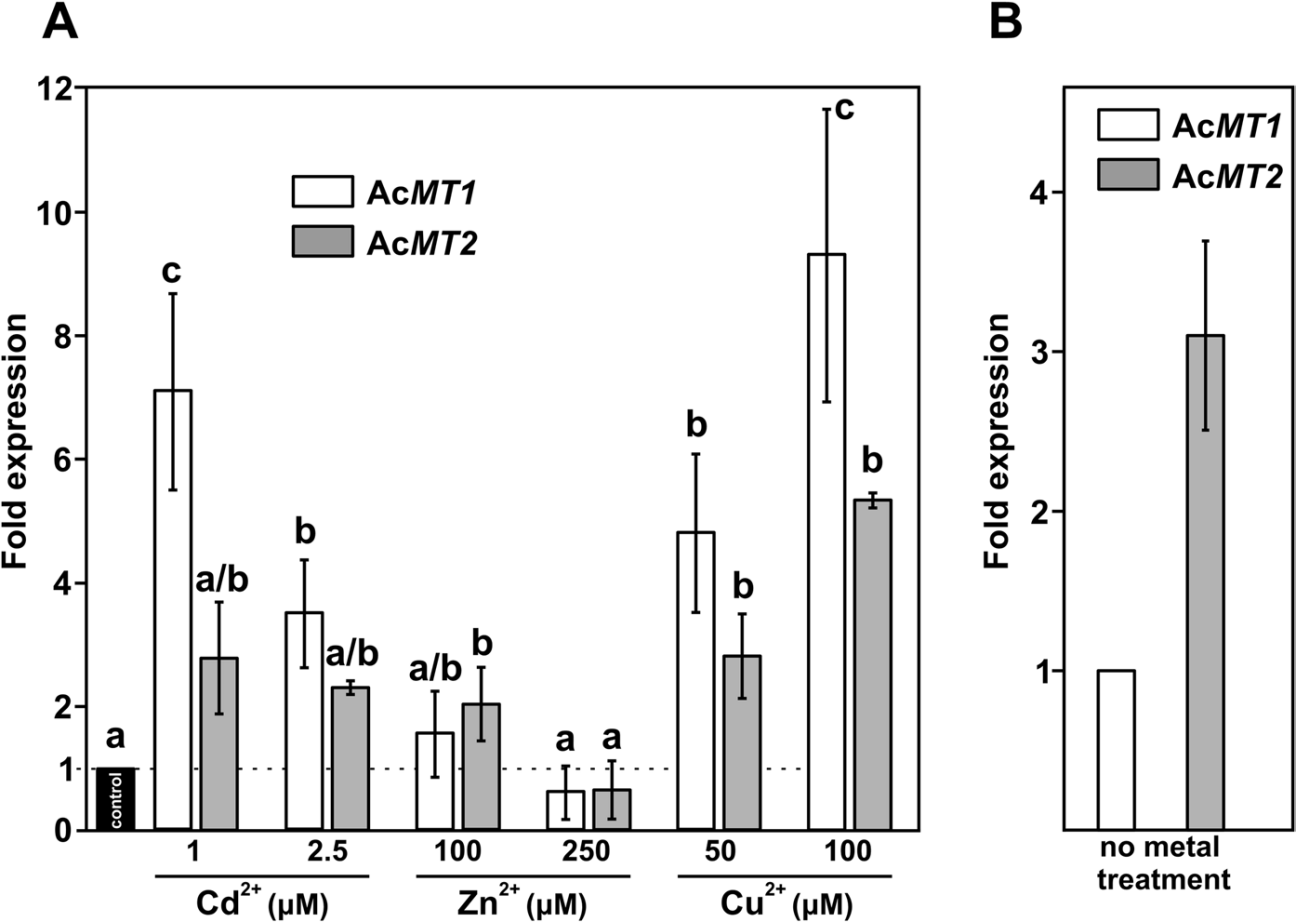
Metal-induced expression of Ac*MT1* and Ac*MT2* in *Agaricus crocodilinus* mycelia. **A** Ac*MT* gene expression was analyzed by qRT-PCR in *A. crocodilinus* mycelia exposed for 24 h to the indicated concentrations of Cd^2^⁺, Zn^2^⁺, or Cu^2^⁺. Relative mRNA levels of Ac*MT1* and Ac*MT2* are shown in comparison to untreated mycelium grown in PD medium (expression set to 1, dashed line). **B** Basal expression of Ac*MT1* and Ac*MT2* in the absence of metal treatment. Transcript levels were normalized to the expression of *A. crocodilinus* β-tubulin (GenBank accession PV055698). Values represent means ± standard deviation from three independent biological replicates. Different letters above the bars indicate statistically significant differences (one-way ANOVA followed by Tukey’s test, p < 0.05)

To confirm that Ac*MT*s are expressed in the mycelia and to see to what extent, the mycelial isolate of *A. crocodilinus* was exposed to elevated metal concentrations for 24 h and qRT-PCR was performed. As a control gene, *A. crocodilinus* β-tubulin was used. As documented in Fig. 7A, the mycelium responded by increasing the transcription of both Ac*MT1* and Ac*MT2* in the presence of excess Cu^2+^, up to 9- and fivefold at the tested conditions, respectively, and of Ac*MT1* upon Cd^2+^ exposure, up to sevenfold, at the tested conditions. The average transcript levels of Ac*MT2* appeared in Cd^2+^ treatments were only marginally elevated (up to threefold) and Zn^2+^ did not induce a significant accumulation of either Ac*MT* transcript under the conditions used. However, the basal expression level of the Ac*MT2* gene is approximately three times higher compared to the basal level of the Ac*MT1* gene (Fig. 7B).

## Discussion

The Cd accumulation phenotype of *A. crocodilinus* has been documented repeatedly (Meisch et al. 1977; Melgar et al. 1998; Cocchi et al. 2006; Cocchi and Vescovi 1997). In our recent study (Sácký et al. 2025), we analyzed a sporocarp specimen with high Cd and Zn content. To avoid toxicity, such metals must be safely sequestered inside fungal cells. In other metal-accumulating fungi, this is typically achieved via MTs, as in Zn/Cd-accumulating *Amanita muscaria* (Sácký et al. 2022) and Cd-accumulating *C. carcharias* (Sácký et al 2021) or metal binding peptides, as seen in Zn-accumulating *Russula* spp. (Leonhardt et al. 2014, 2019). By contrast, peptide-mediated binding appears limited in *Boletus edulis* (Collin-Hansen et al. 2007) and *Thelephora penicillata* (Borovička et al. 2022; 2023), while in *Hebeloma mesophaeum*, MTs are undetectable in sporocarps yet can be upregulated under metal stress in laboratory-grown mycelia (Sácký et al. 2014).

In *A. crocodilinus*, earlier studies suggested that Cd binds weakly to a 10 kDa Cys-free glycoprotein called "mycophosphatin" (Meisch et al. 1983). However, we did not detect this compound in our specimen. Instead, SEC-ICP-MS analysis revealed a distinct 3.4 kDa Cd/Zn-binding complex in sporocarp extracts. Mycelium exposed to Cd showed a minor mycophosphatin-like shoulder, and the 3.4 kDa peak was also prominent. Under Zn exposure, no peaks of expected sizes formed; only a broad > 13.7 kDa signal was observed, likely representing metalated proteins or extraction artifacts, as previously reported for *C. carcharias* (Borovička et al. 2019) and *T. penicillata* (Borovička et al. 2023).

The absence of mycophosphatin and the presence of a distinct low-molecular-weight complex in our sample may reflect ecotypic variation. The population from which the specimen was collected (Morašice) may represent a lineage that does not rely on mycophosphatin for metal detoxification, instead favoring a smaller peptide-based mechanism. Comparable ecotype-specific differences in metal chelation strategies have been reported in *H. mesophaeum* (Sácký et al. 2014) as well as in *Suillus luteus* and *Suillus bovinus* (Colpaert et al. 2011).

To identify the Cd- and Zn-associated ligands, we focused on characterizing the dominant 3.4 kDa peptide complex observed in both sporocarp and Cd-treated mycelium. In organisms without sequenced genomes, protein identification remains challenging. Traditional approaches such as de novo peptide sequencing require both highly purified protein fractions and specialized instrumentation (Liu et al. 2023). To circumvent these limitations, we employed a functional expression strategy: total RNA was isolated from *A. crocodilinus* sporocarps, converted into cDNA, and cloned into a Cd-sensitive *S. cerevisiae* mutant. This strain fails to grow in the presence of 100 µM Cd unless complemented by a Cd-protective gene. Transformants that survived on Cd-containing plates were presumed to express metal-binding proteins. Plasmid recovery and sequencing of these clones yielded two MT-encoding genes, designated Ac*MT1* and Ac*MT2*.

Subsequent MS/MS analysis of the 3.4 kDa SEC sporocarp fractions confirmed the presence of AcMT2, consistent with its role as a Cd-binding peptide. This correlates with gene expression patterns in mycelium, where AcMT2 is detectable even in the absence of metal exposure, suggesting AcMT2 is the main Cd-storage peptide. Although AcMT1 was expressed in the Zn-exposed mycelia, no Zn-AcMT1 complex (expected size 5.1 kDa) was detected during the SEC experiments, neither in sporocarp, nor in mycelia, suggesting AcMT1 may not form stable soluble complexes under our extraction conditions. Given this, we examined the detoxification-related and regulatory features of both proteins in more detail to elucidate their respective roles in metal detoxification.

Although the longer AcMT1 (~ 5.1 kDa) did not yield a Zn-associated peak in SEC of mycelia or sporocarp, its functional importance became evident during cDNA library screening—Ac*MT1* was recovered multiple times, indicating functional transcription in sporocarps. Interestingly, functional assays in metal-sensitive *S. cerevisiae* mutants revealed that AcMT1 consistently outperformed AcMT2 in conferring tolerance to Cd, Zn, and Cu, evident across both plate and IC₅₀ assays. qRT-PCR analysis showed that under metal exposure (Cd or Zn), AcMT1 expression was significantly upregulated compared to AcMT2, whereas under basal conditions, AcMT2 was the dominant transcript. These data suggest that AcMT2 functions as a constitutive metal-binding peptide, while AcMT1 serves as an inducible, high-capacity response to acute metal stress. Metal-specific inducible MTs have been found by several studies, such as Cd/Cu-inducible Pi*MT1* of *Paxillus involutus* (Bellion et al. 2007) or Hc*MT*s of *Hebeloma cylindrosporum* (Ramesh et al. 2009). In fact, Ac*MT1* behaved very similarly to Pi*MT1*, which is almost silent under normal conditions but induced by Cd and Cu, yet not by Zn. Likewise, in *Hebeloma cylindrosporum*, two MT paralogs display distinct induction profiles to Cd and Cu (Ramesh et al. 2009). These parallels validate our model of specialized, metal-specific MT regulation. Concurrent with the low response of mycelial mRNA levels to Zn, mycelial exposure to Zn failed to produce the 3.4 kDa SEC peak, suggesting that Zn detoxification under acute stress may rely on alternative mechanisms, such as export or organellar sequestration, with AcMT1 acting in some other way, such as a transient chaperone or free radical scavenger. The sporocarp 3.4 kDa Zn peak, therefore, may represent either an extraction artifact or an alternative ligand not captured via our cDNA approach.

Phylogenetic and structural analyses suggest that AcMT1 likely represents a derived paralog of AcMT2, originating through an internal domain duplication and reinsertion event within an ancestral metallothionein gene. This mechanism seems to be an imperfect event similar to the tandem duplications observed in the fungal MTs highlighted in this study (Fig. 5 and Table 1). The duplication event is likely universal for MTs of mammals (Moleirinho et al. 2011), squamates (Trinchella et al. 2008), insects (Merritt and Bewick 2017), fish (Bargelloni et al. 1999). A detailed analysis of the underlying gene copy mechanism was performed for the evolution of *Tetrahymena malaccensis* where the longer *TmalaMTT3* gene originated from a shorter *TmalaMTT4* gene duplication (de Francisco et al. 2016). In the case of fungi, a detailed analysis on the MT gene evolution has not yet been published and thus our work is the first to open this question.

**Table 1:** Characteristics of AcMT homologs identified in *Agaricomycetes*

Species (order)	Peptide	Length (AA)	Protection in yeasts			Induction in mycelia			References
			Cd	Zn	Cu	Cd	Zn	Cu	
*Agaricus crocodilinus* (*Agaricales*)	AcMT1^a^	49	+	+	+	+	−	+	This study
	AcMT2^a^	32	+	−	+	−	−	+	
*Amanita strobiliformis* (*Agaricales*)	AsMT2	34	+	−	+	+	−	−	Hložková et al. (2016)
*Cystoderma carcharias* (*Agaricales*)	CcMT1^a^	33	+	+	+	n.d	n.d	n.d	Sácký et al. (2021)
	CcMT2^a^	33	+	−	+	n.d	n.d	n.d	
*Hebeloma cylindrosporum* (*Agaricales*)	HcMT1^a^	59	+	n.d	+	−	n.d	+	Ramesh et al. (2009)
	HcMT2^a^	57	+	n.d	+	+	n.d	+	
*Hebeloma mesophaeum* (*Agaricales*)	HmMT1^a^	59	+	+	+	+	+	n.d	Sácký et al. (2014)
	HmMT2^a^	58	+	−	+	n.d	n.d	n.d	
	HmMT3^a^	52	+	−	+	+	+	n.d	
*Laccaria bicolor* (*Agaricales*)	LbMT1	33	+	n.d	+	+	n.d	+	Liu et al. (2022); Reddy et al. (2014)
	LbMT2a^a^	58	+	n.d	+	+	−	+	
	LbMT2b^a^	58	+	n.d	+	n.d	n.d	n.d	
*Paxillus involutus* (*Boletales*)	PiMT1	34	+	−	+	+	−	+	Bellion et al. (2007)
*Pisolithus albus* (*Boletales*)	PaMT1	35	+	n.d	+	+	n.d	+	Reddy et al. (2016)
*Suillus himalayensis* (*Boletales*)	ShMT1^a^	34	+	+	+	−	n.d	+	Kalsotra et al. (2018)
	ShMT2^a^	34	+	+	+	−	n.d	+	

Data were compiled from published studies. Functional activity in yeast assays (plate spot or IC₅₀ assays) and transcriptional induction in mycelia under metal stress were evaluated. A positive outcome (e.g., metal tolerance or induction) is marked as (+), a lack of effect as (−), and “n.d.” indicates data not available or not determined

^a^Presumably duplicated genes in the designated species

When the genomic and cDNA sequences of Ac*MT1* and Ac*MT2* were inspected in detail, it was found that they are very similar, to the extent of being copies of each other. It was found that the genes are indeed duplicates of each other, however, in a way that a part of the gene was duplicated and then inserted within itself, unlike in other fungi that we surveyed (Fig. 5, Table 1, Supplementary Data 1), where the duplicated gene sequence is inserted next to itself. Internal domain duplications in eukaryotic genes have occurred frequently during the evolution to improve a protein function or to enable a gene to perform a novel function (Li and Makova 2005). Noteworthy, accumulation of multiple, nearly perfect tandem repeats of modules containing 7 Cys have been implicated as adaptive evolutionary mechanism acting on metal detoxification MTs in mollusks (up to 9 Cd-specific repeats; Calatayud et al. 2021) and basidiomycetous yeast *C. neoformans* (3 and 5 Cu-specific repeats; Ding et al. 2013; Palacios et al. 2014a). Adaptive duplication of stress-related genes, which then rarely diverge with respect to general function, but may result in regulatory divergence or adjustment of the substrate specificity, has been implicated as an important defense response of fungi (Wapinski et al. 2007; Emri et al. 2018). The domain duplication of Ac*MT1* may underlie its broader metal-binding capacity and heightened inducibility, features that would confer an adaptive advantage under fluctuating environmental metal pressures. This idea aligns with evolutionary models proposing that MT gene duplications enable functional specialization, allowing different paralogs to be either constitutively expressed or inducibly upregulated in response to acute metal stress as discussed before.

While the coding sequence architecture supports a duplication-based origin, the lack of sequence similarity between introns of Ac*MT2* and Ac*MT1* complicates direct reconstruction of the evolutionary trajectory. Nevertheless, such intronic divergence is not unexpected. In fact, introns of duplicated genes often accumulate point mutations rapidly, which can stabilize the novel gene by disrupting sequence identity and preventing homologous recombination or gene conversion. This mechanism has been proposed as a key step in the fixation of duplicated genes (Micheli and Camilloni 2022), and may have played a role in the divergence of Ac*MT1* from its presumed ancestor.

## Conclusions

*Agaricus crocodilinus* naturally accumulates heavy metals, even in unpolluted environments. A substantial fraction of intracellular Cd is complexed with AcMT2, a constitutively expressed MT abundant in mature sporocarps. In contrast, AcMT1, a closely related paralog, is minimally expressed under basal conditions but is strongly induced in the mycelium in response to acute Cd and Cu, but not Zn, exposure. Despite its inducibility in mycelium and more effective performance in yeast metal-tolerance assays for Cd, Cu, and Zn, AcMT1 does not give rise to a distinct metal–MT complex detectable in sporocarps. This dichotomy suggests functional specialization: AcMT2 likely acts as a baseline metal scavenger, supporting long-term storage or buffering roles, while AcMT1 functions as a rapid-response detoxifier activated under sudden metal stress. Comparative sequence analyses indicate that AcMT1 arose through an internal gene duplication event, followed by regulatory rewiring and subtle structural divergence. Together, these findings illustrate how gene duplication and subsequent neofunctionalization can yield complementary metal-handling strategies in fungi, enabling flexibility in coping with both chronic and acute metal exposure in heterogeneous environments.

## Supplementary Information

Below is the link to the electronic supplementary material.

Supplementary file1 (PDF 1727 KB)

## Data Availability

No datasets were generated or analysed during the current study.
