# Supplementary material for: Identification of two metallothioneins in Agaricus crocodilinus reveals gene duplication and domain expansion, a pattern conserved across fungal species
Source: Biometals. 2025 Jul 18;38(5):1569–85. doi: 10.1007/s10534-025-00721-6 (PMC12508003; doi:10.1007/s10534-025-00721-6)
Supplement: Supplementary file 1 — Supplementary file1 (PDF 1727 KB) [file 10534_2025_721_MOESM1_ESM.pdf]

Identification of two metallothioneins in *Agaricus crocodilinus* reveals gene duplication and domain expansion, a pattern conserved across fungal species

Jan Sácký[1]*, Anna Chaloupecká[1], Jiří Šantrůček, Antonín Kaňa, Tereza Leonhardt, Jan Borovička, Pavel Kotrba

*corresponding author:
email address: sackyj@vscht.cz (Jan Sácký)

[1] These authors contributed equally to this work.

Table S1: Yeast strains used in this study

Table S2: Primers used in this study according to purpose

Figure S1: MS data

Figure S2: Metal dose-dependent growth of metal-sensitive strains of *Saccharomyces cerevisiae*

Data S1: Gene analysis

Supplementary Table S1. *S. cerevisiae* metal-sensitive mutants used in this study.

Sensitivity	Gene Deletion	Strain	Genotype	Source
Cd sensitive	*ycf1*Δ	DTY168	MATα his6 leu2-3,-112 ura3-52 ycf1::hisG	Li, Z.S. et al. 1996
Cu sensitive	*cup1*Δ	DTY113	MATα trp1-1 leu2-3,-112 gal1 ura3 50 cup1Δ61	Tamai, K.T. et al. 1993
Zn sensitive	*zrc1*Δ*cot1*Δ	CM137	MATα can1-100 his3-11,15 leu2-3,112 trp1 1 ura3-52 zrc1::His3 cot1::KanR	MacDiarmid, C.W. et al. 2000

Table S2 Primers used in this study according to purpose.

Primer name	Sequence (5´→3´)	purpose
AcMT1_P426_F	TAGAACTAGTGGATCCATGGGAAGAACCACCATGAA	Insertion of coding AcMT1 to p426GPD
AcMT1_P426_R	GCAGCCCGGGGGATCCTCAACACTTACAACTGCCAGC	Insertion of coding AcMT1 to p426GPD
AcMT2_P426_F	TAGAACTAGTGGATCCATGCACTCTACTCCGATCG	Insertion of coding AcMT2 to p426GPD
AcMT2_P426_R	GCAGCCCGGGGGATCCTCAGCACTTGCATTCGCCAG	Insertion of coding AcMT2 to p426GPD
qAcMT1-F	GGAAGAACCACCATGAACGC	quantitative PCR of AcMT1
qAcMT1-R	CGCATTTGCAGTTATCGCCA	quantitative PCR of AcMT1
qAcMT2-F	TCCGATCGACTACCACGGAG	quantitative of AcMT2
qAcMT2-R	TCAGCACTTGCATTCGCCAG	quantitative of AcMT2
qAcbTub-F	GTGCGGTAACCAAATTGGTGCC	quantitative PCR of Tubulin
qAcbTub-R	GAACTCCATTTCGTCCATACC	quantitative PCR of Tubulin
p416-F	GGTAGGTATTGATTGTAATTCTG	sequencing of inserts in p426GPD
p416-R	GTTGTCTAACTCCTTCCTTTTCG	sequencing of inserts in p426GPD
gAcMT1-F	TTGCAAGCAGTGGTATCAACGC	genomic sequence isolation
gAcMT1-R	TGCAGAGCAGCATAATGAGACC	genomic sequence isolation
gAcMT2-F	CTCAACTTCAATCGCCCTACCA	genomic sequence isolation
gAcMT2-R	CATAAGTGACCACTAGATTCGG	genomic sequence isolation
p426-CL-F	TCTCATCGTACCCCGTCAAGCTTATCGATACCGTCG	linearization of p426GPD
p426-CL-R	CCACTGCTTGCAATGTATCGAATTCCTGCAGCCCG	linearization of p426GPD
3FSCL	CGGGGTACGATGAGACACCATTTTTTTTTTTTTTTTTTTTVN	1st strand cDNA syntesis
3SSCL	CGGGGTACGATGAGACACCA	2st strand cDNA syntesis
5FSCL	GCTAATCATTGCAAGCAGTGGTATCAACGCAGAGTACATrGrGrG	1st strand cDNA syntesis
5SSCL	CATTGCAAGCAGTGGTATCAAC	2st strand cDNA syntesis

Fig. S1 Mass spectrum of the peptide AcMT2 obtained by Parallel Accumulation–Serial Fragmentation Mass Spectrometry (precursor m/z 556.0110, 28.8 eV, retention time 9.4 min, ion mobility $1/K_0 = 0.800$, charge 5+). The black peaks represent signal intensity of detected ions across the m/z range. Fragment ions from collision-induced dissociation are annotated as b-ions (red, originating from the N-terminus) and y-ions (blue, originating from the C-terminus). The most probable precursor sequence match was a fragment of AcMT2 (75% sequence coverage) of theoretical mass 2775,0168, experimentally determined as 2775,015 (Fragmentation resulted in the detection of six ions, with four (y11, y13, y14, y15) forming a continuous C-terminal sequence corresponding to the amino acids N–C–G–D. The first eight amino acids from the N-terminus were not recovered.

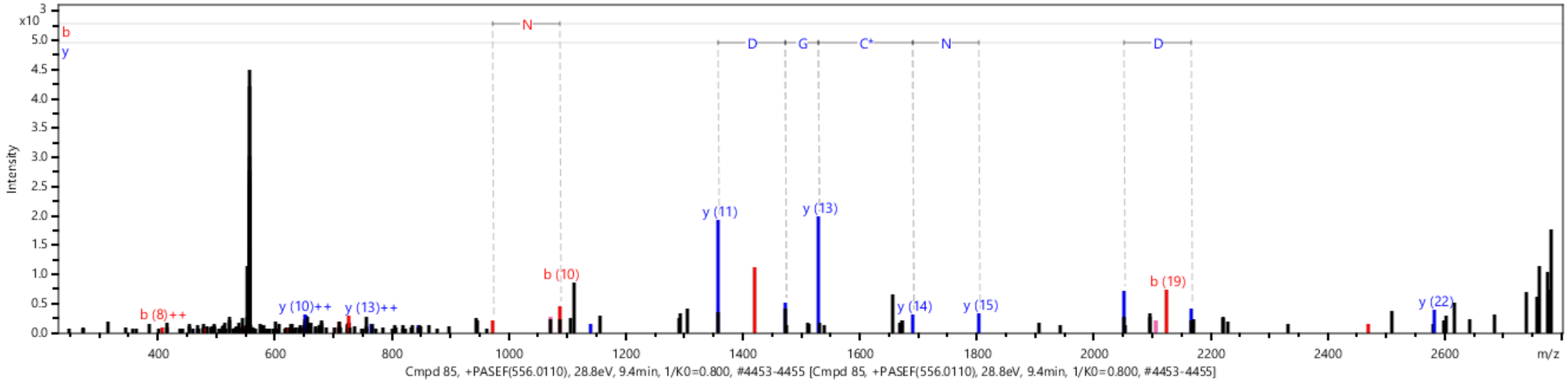

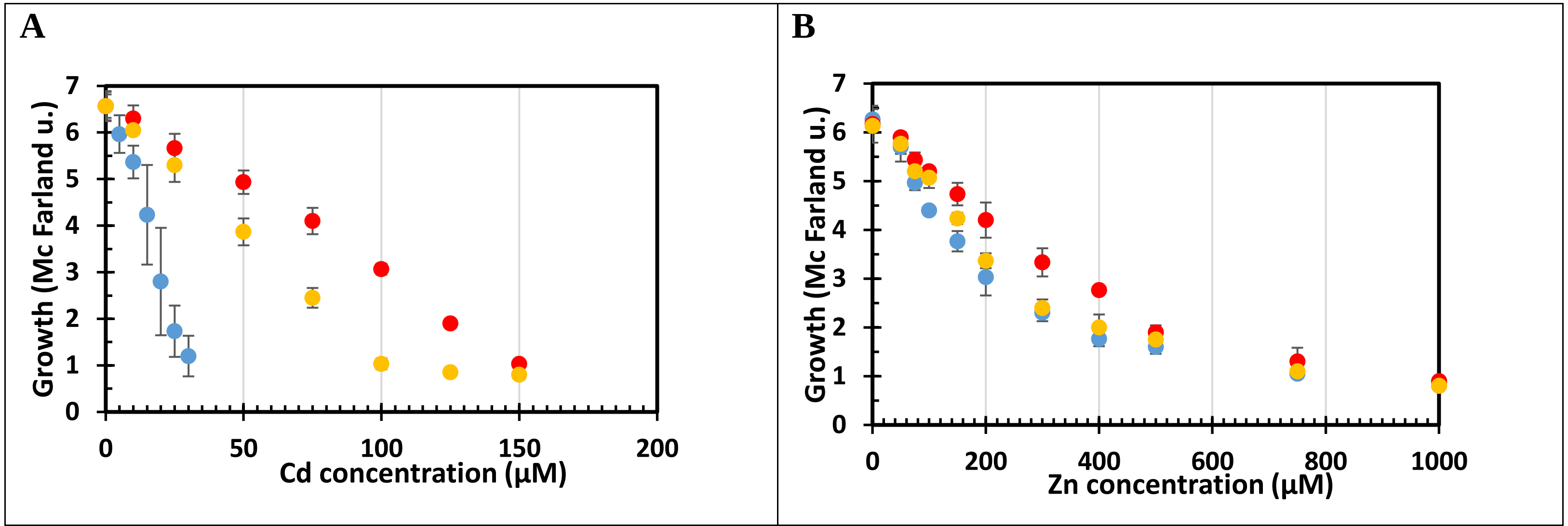

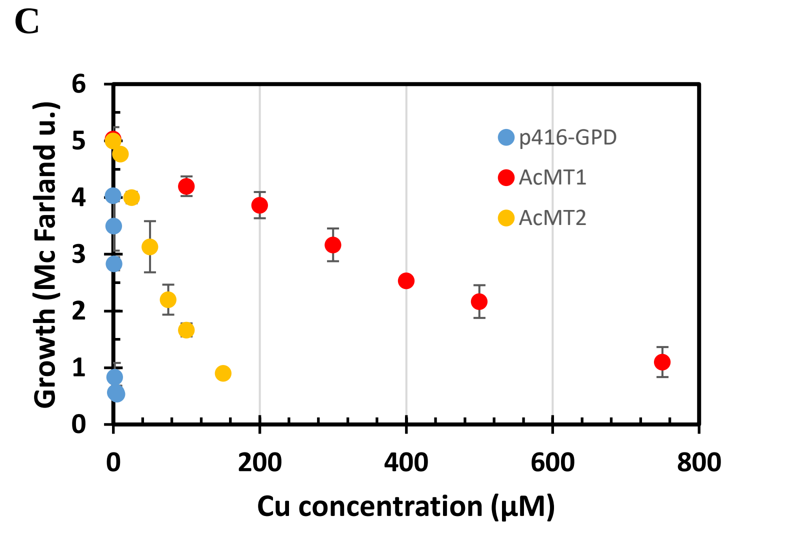

Fig. S2. Metal dose-dependent growth of metal-sensitive strains of *Saccharomyces cerevisiae* transformed with empty p426GPD expression vector (●) and/or p426GPD inserted with Ac*MT1* (●) or Ac*MT2* (●) cDNAs. Individual transformants of Cd-sensitive *ycf1*Δ (**A**), Zn-sensitive *zrc1*Δ*cot1*Δ (**B**), and Cu-sensitive *cup1*Δ (**C**) yeasts were grown in liquid SD medium with different concentrations of Cd^{2+}, Zn^{2+}, or Cu^{2+}. The turbidity of the culture was measured after 24 h (*ycf1*Δ and *zrc1*Δ*cot1*Δ cells), or 48 h (*cup1*Δ cells). Plotted values represent an average of three independent replicates ± standard deviation of the mean.

Supplementary Data S1: Gene analysis

HcMT1

MQFTSILVNQACGSDNCQCDAACTCSSGSCHAPVNRACGSSDCNCNSSCGCESNNCNCN

ATGCAATTCACTTCTATCCTCGTCAACCAAGCTTGCGGTTCTGACAATTGCCAGTGCGACGCAGCTTGCACCTGC
TCCTCTGGCTCTTGCCACGCCCCCGTCAACCGCGCTTGCGGTTCCAGCGACTGCAACTGCAACAGCTCGTGCGGC
TGCGAGTCCAACAACTGCAACTGCAACTGA

ATGCAATTCGTGAGTTTACTCCTTACCTTACCACTGCTCTACACTGACCATCGGTAGACTTCTATCCTCGTCAAC
CAAGCTTGCGGTTCTGACAATTGCCAGTGCGACGCAGCGTACGTCATATCACCTTCTGTTATTGCCAACTTGCGG
CTAACAAGTTAACTCTTTCATTACCCAGTTGCACCTGCTCCTCTGGCTCTTGCCACGCCCCCGTCAACCGCGCTT
GCGGTTCCAGCGACTGCAACTGCAACAGCTCGTACGCATTACCACTTTTGTGTCATATTTAACCGTCTAATAATT
TTGTCCTCGTCATCCATCAGGTGCGGCTGCGAGTCCAACAACTGCAACTGCAACTGA

DNA

```
A       GTCAACCAAGCTTGCGGTTCTGACAATTGCCAGTGCGACGCAGCGTACGTCATATCACCT  60
B       GTCAACCGCGCTTGCGGTTCCAGCGACTGCAACTGCAACAGCTCGTACGCATTACCACTT  60
        *******  ***********   * * *** * *** **    ******   ** *** *

A       TCTGTTATTGCCAACTTGCGGCTAACAAG-TTAACTCTTTCATTACCCAGTTGCACCTGC  119
B       TTGTGTCATATTTAACCG--TCTAATAATTTTGTCCTCGTCATCCATCAGGTGCGGCTGC  118
        *    *  *    *   *   **** **  **  *    ****    *** ***  ****

A       TCCTCTGGCTCTTGCCACGCCCCC         143
B       GAGTCCAACAACTGCAACTGCAAC         142
           **   *   *** **  *  *
```

cDNA

```
A       GTCAACCAAGCTTGCGGTTCTGACAATTGCCAGTGCGACGCAGCTTGCACCTGCTCCTCT  60
B       GTCAACCGCGCTTGCGGTTCCAGCGACTGCAACTGCAACAGCTCGTGCGGCTGCGAGTCC  60
        *******  ***********   * * *** * *** **    * ***  ****   **

A       GGCTCTTGCCACGCCCCC      78
B       AACAACTGCAACTGCAAC      78
          *   *** **  *  *
```

Protein

```
A       VNQACGSDNCQCDAACTCSSGSCHAP      26
B       VNRACGSSDCNCNSSCGCESNNCNCN      26
        ** ****  * *   * * *  *
```

HcMT2

MQIVQNSLVSQSSGCTCTSCKCGSNCTCGAPVNQSSGCGSSSCTCTSCTCKAGECKC

ATGCAGATCGTTCAAAACAGTCTCGTCTCCCAGTCTTCTGGGTGCACCTGTACTTCCTGCAAGTGCGGCTCTAAC
TGCACCTGCGGAGCCCCTGTCAACCAGTCCTCTGGATGCGGAAGCTCTTCCTGCACCTGCACTTCCTGCACCTGC
AAGGCTGGCGAGTGCAAATGCTAA

ATGCAGGTCCGTATTTCCTTCCCCGGCCTTGGACATCGTGCTCATCTCACCGTGCGTTCCCAACAGATCGTTCAA
AACAGTCTCGTCTCCCAGTCTTCTGGGTGCACCTGTACTTCCTGCAAGTGCGGCTCTAAGTAGGTTTCCCCCAGG
TCCCCCCTTCGTTGGATGCTGACGATAGATGTGATTCTTAGCTGCACCTGCGGAGCCCCTGTCAACCAGTCCTCT
GGATGCGGAAGCTCTTCCTGCACCTGCACTTTGTGAGTAGTTCGTCATCTCGCGGTATCTGGTGTTCTGACGTCG
GTGATCTTCCCTAGCTGCACCTGCAAGGCTGGCGAGTGCAAATGCTAA

DNA

```
A       CAGTCTTCTGGGTGCACCTGTACTTCCTGCAAGTGCGGCTCTAAGTAGGTTTCCCCCAGG  60
B       CAGTCCTCTGGATGCGGAAGCTCTTCCTGCACCTGCACTTC---GTGAGTAGTTCGTCAT  57
        ***** ***** ***    *  *********  ***   **   **  **    *

A       TCCCCCCTTCGTTGGATGCTGACGATAGATGTGATTCTTAGCTGCACCTGC   111
B       CTCGCGGTATCTGGTGTTCTGACGTCGGTGATCTTCCCTAGCTGCACCTGC   108
          * *  *   * *  * ******   *   *  * * *************
```

cDNA

```
A       CAGTCTTCTGGGTGCACCTGTACTTCCTGCAAGTGCGGCTCTAACTGCACCTGC 54
B       CAGTCCTCTGGATGCGGAAGCTCTTCCTGCACCTGCACTTCC---TGCACCTGC 51
        ***** ***** ***    *  *********  ***   **    *********
```

Protein

```
A       QSSGCTCTSCKCGSNCTC      18
B       QSSGCGSSSCTCTS CTC      17
        *****    * * * ***
```

HmMT1

MQFTSTLVNQA**C**GSAN**C**S**C**DSS**C**T**C**SSGS**CH**A**P**VNQA**C**GSSS**C**N**C**NSS**C**G**C**DSNN**C**N**C**S

ATGCAATTCACTTCCACCCTCGTCAACCAAGCTTGCGGCTCAGCCAACTGCAGCTGCGACTCGAGCTGCACCTGC
TCGTCCGGCTCTTGCCACGCCCCCGTCAACCAGGCATGCGGTTCAAGCTCCTGCAACTGCAACAGCTCCTGTGGC
TGCGACTCCAACAACTGCAACTGCTCTTGA

ATGCAATTCGTCAGCTTACCCCTACCTTGCCACTGTTCTACGCTAACCTTCGCTCCATTAGACTTCCACCCTCGT
CAACCAAGCTTGCGGCTCAGCCAACTGCAGCTGCGACTCGAGGTACGCCGTGTCACCTTTTTCATTGCCAACGTG
CCGCTAATAACTATCTTCATCACCAGCTGCACCTGCTCGTCCGGCTCTTGCCACGCCCCCGTCAACCAGGCATGC
GGTTCAAGCTCCTGCAACTGCAACAGCTCGTACGCCTTTCGACTTTTATACCACTCTGAACCGTCTAATAATTTT
GTCCTCGTCACTAGCTGTGGCTGCGACTCCAACAACTGCAACTGCTCTTGA

DNA

```
A       GTCAACCAAGCTTGCGGCTCAGCCAACTGCAGCTGCGACTCGAGGTACGCCGTGTCACCT  60
B       GTCAACCAGGCATGCGGTTCAAGCTCCTGCAACTGCAACAGCTCGTACGCCTTTCGACTT  60
        ******** ** ***** ***  *  ***** **** **     ******* *   ** *

A       TTTTCATTGCCAACGTGCCGCTAATAA--CTATCTTCATCACCAGCTGCACCTGCTCGTC  118
B       TTATACCACTC-TGAACCGTCTAATAATTTTGTCCTCGTCACTAGCTGTGGCTGCGACTC  119
        ** *      *      *  *******   * ** ** **** *****   ****   **

A       CGGCTCTTGCCACGCCCCC     137
B       CAACAACTGCAACTGCTCT     138
        *  *   *** **  * *
```

cDNA

```
A       GTCAACCAAGCTTGCGGCTCAGCCAACTGCAGCTGCGACTCGAGCTGCACCTGCTCGTCC  60
B       GTCAACCAGGCATGCGGTTCAAGCTCCTGCAACTGCAACAGCTCCTGTGGCTGCGACTCC  60
        ******** ** ***** ***  *  ***** **** **     ***   ****   ***

A       GGCTCTTGCCACGCCCCC      78
B       AACAACTGCAACTGCTCT      78
          *   *** **  * *
```

protein

```
A       VNQACGSANCSCDSSCTCSSGSCHAP      26
B       VNQACGSSSCNCNSSCGCDSNNCNCS      26
        *******  * * *** * *  *
```

HmMT2

MQIVQNTLVSRTRTPDCTCGTCECAPTCTCAAPVNQSGCGSSSCTCTSCACKPGECKC

ATGCAGATCGTTCAAAACACCCTCGTCTCCCGGACTCGGACTCCTGATTGTACCTGCGGCACCTGCGAGTGCGCC
CCTACCTGCACCTGCGCAGCCCCTGTCAACCAGTCTGGATGCGGCAGCTCTTCCTGCACCTGTACTTCCTGTGCC
TGCAAGCCTGGCGAGTGCAAATGCTAA

ATGCAGGTGGGTGTCTCCTTCCTTGCCTTTGAACATCGTGCTCATCGCATTGCTCGTTCCCAATAGATCGTTCAA
AACACCCTCGTCTCCCGGACTCGGACTCCTGATTGTACCTGCGGCACCTGCGAGTGCGCCCCTACGTAGGTTCCT
CTCTATGTTCCCCTCGTCGTGGATACTGATGATATCTTTGGTCCTTAGCTGCACCTGCGCAGCCCCTGTCAACCA
GTCTGGATGCGGCAGCTCTTCCTGCACCTGTACTTCGTGAGTAGTTCGCTGTTCTTCGAGTGCACTTCCCTGTAT
CCTAATATCGGTGATAACCATAGCTGTGCCTGCAAGCCTGGCGAGTGCAAATGCTAA

DNA

```
A       GATTGTACCTGCGGCACCTGCGAGTGCGCCCCTAC---GTAGGTTCCTCTCTATGTTCC-   56
B       CAGTCTGGATGCGGCAGCTCTTCCTGCACCTGTACTTCGTGAGTAGTTCGCTGTTCTTCG   60
         * * *   ******* **     *** **  ***    *  **   ** ** *  * *

A       ---CCTCGTCGTGGATACTGATGATATCTTTGGTCCTTAGCTGCACCTGC    103
B       AGTGCACTTCCCTGTATCCTAATATCGGTGATAACCATAGCTGTGCCTGC    110
            * * **   *   *  *  **   *     ** ******  *****
```

cDNA

```
A       GATTGTACCTGCGGCACCTGCGAGTGCGCCCCTAC---CTGCACCTGC      45
B       CAGTCTGGATGCGGCAGCTCTTCCTGCACCTGTACTTCCTGTGCCTGC      48
         * * *   ******* **     *** **  ***   ***  *****
```

Protein

```
A       DCTCGTCECAPT CTC        15
B       QSGCGSSSCTCTSCAC        16
           **   *  * * *
```

HmMT3

MQIVQKSSECTCDPCECGANCTCAAPVNQSSGCGSSSCTCTSCACKPGECKC

ATGCAGGTGGGTGTCTCCTTCCTTGCCTTTGAACATCGTGCTCATCGCATCGCTCGTTCCCAATAGATCGTTCAA
AAGTCTTCTGAATGCACCTGCGACCCCTGCGAGTGCGGCGCTAAGTAGGTTCCTCTCTATGTTCCCCTCGTCGCG
GATACTGATGATATCTCTCATCCTTAGCTGCACCTGCGCAGCCCCTGTCAACCAGTCCTCTGGATGCGGCAGCTC
TTCCTGCACCTGTACTTCGTGAGTAGTTCGTTGTTCTTCGAGTGCACTTCCCTGTATCCTAATATCGGTGATAAC
CATAGCTGTGCCTGCAAGCCTGGCGAGTGCAAATGTTAA

ATGCAGATCGTTCAAAAGTCTTCTGAATGCACCTGCGACCCCTGCGAGTGCGGCGCTAACTGCACCTGCGCAGCC
CCTGTCAACCAGTCCTCTGGATGCGGCAGCTCTTCCTGCACCTGTACTTCCTGTGCCTGCAAGCCTGGCGAGTGC
AAATGTTAA

DNA

```
A       AAGTCTTCTGAATGCACCTGCGACCCCTGCGAGTGCGGCGCTAAGTAGGTTC-CTCTCTA  59
B       CAGTCCTCTGGATGCGGCAGCTCTTCCTGCACCTGTACTTCGTGAGTAGTTCGTTGTTCT  60
         **** **** ****  * **    *****   **     *       ****  * *

A       TGTTCCCCTCGTCGCGGATACTGATGATATCTCTCATCCTTAGCTGCACCTGC 112
B       TCGAGTGCACTTCCCTGTATCCTAATATCGGTGATAACCATAGCTGTGCCTGC 113
        *      * * ** * *   *  *  **   *   * ** ******  *****
```

cDNA

```
A       AAGTCTTCTGAATGCACCTGCGACCCCTGCGAGTGCGGCGCTAACTGCACCTGC        54
B       CAGTCCTCTGGATGCGGCAGCTCTTCCTGCACCTG---TACTTCCTGTGCCTGC        51
         **** **** ****  * **    *****   **     **  ***  *****
```

protein

```
A       KSSECTCDPCECGANCTC      18
B       QSSGCGSSSCTCTS CAC      17
        ** *    * *   * *
```

>LbMT2a
MLFNTLTPISRASSTGCCCTSCKCTSCTCGTAPVNEAGCGSTTCNCTNCACKPEECKC

DNA

ATGCTCTTTGTGAGTACCATCGGTTACGATCTTTCGAATATGTTGCCTAACTTTCTTGCGATTTAGAACACCTTG
ACCCCTATCTCTCGAGCTTCTTCCACGGGATGCTGCTGCACGTCGTGCAAGTGCACGAGGTAAGCCGGAACTACC
TTCTTTTTTTTGGGGGAGATTTCGATGTCTGATGTATGTTTCTTGTAGCTGCACATGCGGGACCGCCCCTGTTAA
TGAGGCAGGATGCGGAAGCACAACCTGCAACTGCACCAAGTGAGTCGCTGTGTTGTCGGTGGAACACTAGTTGCT
GATGAACTTCCAGCTGCGCGTGCAAGCCTGAGGAATGCAAGTGTTAA

ATGCTCTTTAACACCTTGACCCCTATCTCTCGAGCTTCTTCCACGGGATGCTGCTGCACGTCGTGCAAGTGCACG
AGCTGCACATGCGGGACCGCCCCTGTTAATGAGGCAGGATGCGGAAGCACAACCTGCAACTGCACCAACTGCGCG
TGCAAGCCTGAGGAATGCAAGTGTTAA

DNA

```
A       GCTTCTTCCACGGGATGCTGCTGCACGTCGTGCAAGTGCACGAGGTAAGCCGGAACTACC 60
B       GTTAATGAGGCAGGATGCGGAAGCACAACCTGCAACTGCACCAAGTGAGTCGCTGTGT-- 58
        * *  *    * ****** *  ****  * ***** ***** * ** ** **

A       TTCTTTTTTTTGGGGGAGATTTCGATGTCTGATGTATGTTTCTTGTAGCTGCACATGCGG 120
B       ---------TGTCGGTGGAACACTAGTTGCTGATG-----AACTTCCAGCTGCGCGTGCAA 105
                * * ** *  *      *  ******       ***  ****** * ***

A       GACC      124
B       GCCT      109
        * *
```

cDNA

```
A       GCTTCTTCCACGGGATGCTGCTGCACGTCGTGCAAGTGCACGAGCTGCACATGCGGGACC  60
B       GTTAATGAGGCAGGATGCGGAAGCACAACCTGCAACTGCACCAACTGCGCGTGCAAGCCT  60
        * *  *    * ****** *  ****  * ***** ***** * **** * ***  * *
```

protein

```
A       ASSTGCCCTSCKCTSCTCGT    20
B       VNEAGCGSTTCNCTNCACKP    20
            **  * * ** * *
```

LbMT2b

MLFNTFTPISRVSSTGCSCTSCKCTSCTCGTAPVNEAGCGSTTCNCTNCACKPEECKC

ATGCTCTTTAATACCTTCACCCCTATCTCTCGAGTTTCTTCTACAGGATGCTCCTGCACGTCGTGCAAGTGCACC
AGCTGCACCTGCGGGACCGCCCCTGTTAACGAGGCAGGATGTGGAAGCACAACCTGCAATTGCACCAACTGCGCG
TGCAAGCCTGAGGAATGCAAGTGCTAA

ATGCTCTTTGTGAGTACATCGGTTACGATCTTTTGAATATGCCTAACTTTCTTGCGATTTAGAATACCTTCACCC
CTATCTCTCGAGTTTCTTCTACAGGATGCTCCTGCACGTCGTGCAAGTGCACCAGGTAAGCCTAAATTACTTATT
TTTTTTGGAGATTTTGATGTCTGATTTATGTTTCTTGTAGCTGCACCTGCGGGACCGCCCCTGTTAACGAGGCAG
GATGTGGAAGCACAACCTGCAATTGCACCAAGTGAGTCGCTGCTTTGTCGGTGGAAGACTAGTTATAATTACTGA
TGAATTTCATTCAGCTGCGCGTGCAAGCCTGAGGAATGCAAGTGCTAA

DNA

```
A       GTTTCTTCTACAGGATGCTCCTGCACGTCGTGCAAGTGCACCAGGTAAGCCTAAATTACT  60
B       GTTAACGAGGCAGGATGTGGAAGCACAACCTGCAATTGCACCAAGTGAGTCGCTGCTTTG  60
        ***       *******     ****  * ***** ******* ** ** *     *

A       TATTTTTTTTGGAGATTTTGATGTCTGATTTATGTTTCTTGTAGCTGCACCTGCGGGACC  120
B       TCGGTGGAAGACTAGTTATAATTACTGAT--GAATTTCATTCAGCTGCGCGTGCAAGCCT  118
        *   *          ** * **  *****     **** *  ****** * ***  * *
```

cDNA

```
A       GTTTCTTCTACAGGATGCTCCTGCACGTCGTGCAAGTGCACCAGCTGCACCTGCGGGACC  60
B       GTTAACGAGGCAGGATGTGGAAGCACAACCTGCAATTGCACCAACTGCGCGTGCAAGCCT  60
        ***       *******     ****  * ***** ******* **** * ***  * *
```

protein

```
A       VSSTGCSCTSCKCTSCTCGT    20
B       VNEAGCGSTTCNCTNCACKP    20
        *   **  * * ** * *
```

AcMT1

MGRTTMNAIFDIVVDQACSSGNCKCDKCSGNANCNCGDNCKCEAGSCKC

ATGGGAAGAACCACCATGAACGCTGTAGGTGATCAAAATGTTCATGGTAGCAGATGACACTGACCTTCATAATGA
AAGATCTTTGATATTGTCGTCGACCAAGCTTGCTCTAGCGGCAACTGTAAATGTGACAAATGCAGCGGGAACGCT
AAGTGAGTCTGGAATAATCCATACACTGTTCTTGTACTGACAACGCAATGATATTCTAGCTGCAACTGTGGCGAT
AAGTGAGTCTGAACCCCGTATACTGTTCCTGTCCTGACAACAAGATGATATTCCAGCTGCAAATGCGAGGCTGGC
AGTTGTAAGTGTTGA

ATGGGAAGAACCACCATGAACGCTATCTTTGATATTGTCGTCGACCAAGCTTGCTCTAGCGGCAACTGTAAATGT
GACAAATGCAGCGGGAACGCTAACTGCAACTGTGGCGATAACTGCAAATGCGAGGCTGGCAGTTGTAAGTGTTGA

DNA

```
A       TGCTCTAGCGGCAACTGCAACTGTGGCGATAAGTGAGTCTG---AACCCCGTATACTGTT  57
B       TGTAAATGTGACAAATGCAGCGGGAACGCTAAGTGAGTCTGGAATAATCCATACACTGTT  60
        **      * * *** **** * *    ** ************     *   ** ** ******

A       CCTGTCCTGACAACAAGATGATATTCCAGC 87
B       CTTGTACTGACAACGCAATGATATTCTAGC 90
        * *** ********    ********* ***
```

cDNA

```
1       TGTAAATGTGACAAATGCAGCGGGAACGCTAAC       33
2       TGCTCTAGCGGCAACTGCAACTGTGGCGATAAC       33
        **      * * *** **** * *    ** ****
```

Protein

```
A       CSSGNCNCGDN     11
B       CKCDKCSGNAN     11
        *     *    *
```

>HcMT1
MQFTSILVNQACGSDNCQCDAACTCSSGSCHAPVNRACGSSDCNCNSSCGCESNNCNCN
>HcMT2
MQIVQNSLVSQSSGCTCTSCKCGSNCTCGAPVNQSSGCGSSSCTCTSCTCKAGECKC
>HmMT1
MQFTSTLVNQACGSANCSCDSSCTCSSGSCHAPVNQACGSSSCNCNSSCGCDSNNCNCS
>HmMT2
MQIVQNTLVSRTRTPDCTCGTCECAPTCTCAAPVNQSGCGSSSCTCTSCACKPGECKC
>HmMT3
MQIVQKSSECTCDPCECGANCTCAAPVNQSSGCGSSSCTCTSCACKPGECKC
>LbMT2a
MLFNTLTPISRASSTGCCCTSCKCTSCTCGTAPVNEAGCGSTTCNCTNCACKPEECKC
>LbMT2b
MLFNTFTPISRVSSTGCSCTSCKCTSCTCGTAPVNEAGCGSTTCNCTNCACKPEECKC
>AcMT1
MGRTTMNAIFDIVVDQACSSGNCKCDKCSGNANCNCGDNCKCEAGSCKC
